# Comparative analysis of machine learning techniques for temperature and humidity prediction in photovoltaic environments

**DOI:** 10.1038/s41598-025-98607-7

**Published:** 2025-05-05

**Authors:** Montaser Abdelsattar, Ahmed AbdelMoety, Ahmed Emad-Eldeen

**Affiliations:** 1 Electrical Engineering Department, Faculty of Engineering, South Valley University, Qena, 83523 Egypt; 2 Renewable Energy Science and Engineering Department, Faculty of Postgraduate Studies for Advanced Sciences (PSAS), Beni-Suef University, Beni-Suef, 62511 Egypt

**Keywords:** Machine learning, Temperature prediction, Humidity prediction, Photovoltaic environments, Data-Driven forecasting, Energy science and technology, Engineering

## Abstract

This research conducts a comparative analysis of nine Machine Learning (ML) models for temperature and humidity prediction in Photovoltaic (PV) environments. Using a dataset of 5,000 samples (80% for training, 20% for testing), the models—Support Vector Regression (SVR), Lasso Regression, Ridge Regression (RR), Linear Regression (LR), AdaBoost, Gradient Boosting (GB), Decision Tree (DT), Random Forest (RF), and eXtreme Gradient Boosting (XGBoost)—were evaluated based on Mean Absolute Error (MAE), Root Mean Squared Error (RMSE), and the Coefficient of Determination (R²). For temperature prediction, XGBoost demonstrated the best performance, achieving the lowest MAE of 1.544, the lowest RMSE of 1.242, and the highest R² of 0.947, indicating strong predictive accuracy. Conversely, SVR had the weakest performance with an MAE of 4.558 and an R² of 0.674. Similarly, for humidity prediction, XGBoost outperformed other models, achieving an MAE of 3.550, RMSE of 1.884, and R² of 0.744, while SVR exhibited the lowest predictive power with an R² of 0.253. This comprehensive study serves as a benchmark for the application of ML models to environmental prediction in PV systems, a research area that is relatively important. Notably, the results underscore the performance advantage of ensemble-based approaches, especially for XGBoost and RF compared to simpler, linear-based methods such as LR and SVR, when it comes to well-dispersed environmental interactions. The proposed machine-learning based power generation analysis approach shows significant improvements in predictive analytics capabilities for renewable energy systems, as well as a means for real-time monitoring and maintenance practices to improve PV performance and reliability.

## Introduction

As a fundamental component of renewable energy production, Photovoltaic (PV) systems have been introduced as a sustainable and eco-friendly substitute for traditional fossil fuels^1^. Solar PV systems capture the energy of the sun and convert it into electricity using semiconductor materials; they are essential in the move to more sustainable energy sources^2^. Due to the growing demand of renewable energy; it is more important to improve the PV systems’ performance and efficiency^3–6^. The energy production of these systems can be optimized not only to improve their economic feasibility, but also to help meet the goal of reducing carbon emissions and addressing climate change.

The external factors such as temperature and humidity greatly affect PV systems^7^. The electrical properties of PV cells may be influenced by temperature variations and this may affect their efficiency in converting sunlight to energy^8^. However, high temperatures can reduce the effectiveness of PV cells by increasing their internal resistance, and hence lower the energy production^9^. The performance of PV systems may be affected by humidity levels, leading to condensation, corrosion or deterioration of the materials the panels are made of^10^. Temperature and humidity play a significant role in the optimal operation and maintenance of PV systems^11^, and therefore precise temperature and humidity forecasting is a necessity. Consequently, anticipating these environmental elements in advance will allow system configurations to be altered, maintenance tasks to be planned and precautionary measures applied to extend the life of the PV systems and ensure a constant energy output^12^.

As the complexity and variability of climatic conditions have grown, there has been an increasing reliance on Machine Learning (ML) methods for predicting critical parameters affecting the efficiency of renewable energy systems, including PV systems^13^. This is where ML models are very well suited for this purpose because they can analyze huge datasets, detect patterns and establish relationships between input variables and outputs without requiring explicit programming^14^. In the field of renewable energy ML is important for precise and automated temperature and humidity forecasting. These are the factors that are necessary to maximize the efficiency of PV systems^15^. With the help of algorithms that can be adjusted to the ever changing environment, real time monitoring and forecasting is enabled by ML in PV systems^16^. The level of automation in these systems increases operating efficiency, reduces downtime, and results in more uniform energy output^17^. However, temperature and humidity forecasting in PV conditions is not trivial^18^. The energy production of PV systems has intricate and non-linear connections with temperature and humidity, which are environmental factors^19^. The accurate prediction of these correlations is difficult because of a number of factors, including geographic location, seasonal fluctuations, and local meteorological conditions^20^. Moreover, temperature and humidity usually vary simultaneously, making the prediction modeling more complicated^21^. To provide accurate predictions, ML models need to include these ever changing interactions and at the same time reduce errors^22^. Such a high degree of accuracy can only be achieved with sophisticated algorithms that can deal with complex data, and many environmental factors. This highlights why it is important to pick the right ML methods for this particular use case^14^.

The reason for the comparative analysis of several ML techniques for temperature and humidity prediction is the imperative to improve the efficiency of PV systems. Different ML models may or may not be more or less effective in predicting environmental factors depending on the use case and the complexity of the data. Most previous research has been devoted to applying different models to different objectives of renewable energy forecast. However, the literature currently in publication lacks information on the best ML techniques to forecast temperature and humidity in PV environments. Therefore, to choose the most accurate and reliable models for this goal, a thorough review of various ML techniques is required. The accuracy and reliability of environmental forecasts is highly dependent on the choice of the ML model. Each algorithm has its own advantages and disadvantages, especially when it comes to their ability to deal with nonlinear relationships, feature interactions and unexpected data. Inaccurate predictions can result in ineffective management of solar power plants, which in turn will decrease energy output and increase stress on system components. As such, proper model selection is very important to improve prediction accuracy and to ensure long term efficiency and sustainability of PV systems. This research aims to determine the best models to improve decision making in PV system operations. This will enable increased energy output, timely maintenance, and more precise projections.

Motivated by missing a comprehensive comparison of all ML algorithms for temperature and humidity prediction in PV applications, this study provides a thorough comparison of eight common ML algorithms. In essence, the study aims at pursuing the most accurate and reliable models for predicting these essential climatic parameters that directly influence the PV systems performance and effectiveness. To this end explicitly, a wide range of ML techniques including Support Vector Regression (SVR), Linear Regression (LR), Ridge Regression (RR), Lasso Regression, Decision Tree (DT), Random Forest (RF), AdaBoost, Gradient Boosting (GB), and eXtreme Gradient Boosting (XGBoost) are examined. The rationale for selecting these models is identified on a broad range of approaches, simple LR methods and ensemble and boosting methods also. Among them, three primary measurement metrics were applied to compare the performance of each model, as follows: The Coefficient of Determination (R^2^), the Root Mean Squared Error (RMSE), and the Mean Absolute Error (MAE). These metrics offer a comprehensive evaluation of each model’s effectiveness in predicting temperature and humidity, allowing for a direct comparison of the models’ effectiveness in forecasting temperature and humidity in the specific context of PV environments through their error minimization capabilities and goodness of fit to the data.

This research contributes to the field of renewable energy by identifying the most effective ML models for predicting critical environmental parameters, including temperature and humidity, in PV systems. In this investigation, numerous ML algorithms, such as SVR, LR, Ridge, Lasso, DT, RF, AdaBoost, GB, and XGBoost, have been compared and contrasted to provide valuable insights into their respective advantages and disadvantages. The findings contribute to the expanding body of knowledge on the application of ML in PV systems and offer additional insight into the algorithms that are most effective for optimizing environmental forecasts. This research bridges the divide between the operational requirements of PV systems and the selection of ML models to enhance the overall performance and sustainability of PV systems. The practical implications of this research are extensive. Temperature and humidity are crucial for accurate forecasting, as they enable system administrators to optimize energy production by adjusting the settings in accordance with the anticipated conditions. Furthermore, the scheduling of maintenance duties is more effective due to the improved accuracy of environmental forecasts, which in turn delays the deterioration of the system and extends the lifespan of PV infrastructure. This research enhances the predictive capabilities of renewable energy systems and contributes to the overarching objectives of energy production optimization and the promotion of sustainable, data-driven solutions in the renewable energy sector.

**Fig. 1:**
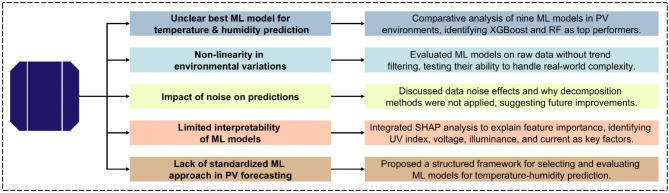
Key challenges and contributions in ML-Based temperature and humidity prediction for PV environments.

This study conducts a comparative analysis of multiple ML models to address critical challenges in temperature and humidity prediction for PV environments. The major challenges encountered in this domain and the corresponding contributions of this research are structured in Fig. 1. This research is remarkable for pointing out the most accurate ML models and evaluating their potential to fit non-linear environmental variability while investigating data noise impacts on prediction performance. In addition, Shapley Additive Explanations (SHAP) analysis can be used to increase the interpretability of the model, allowing for a better understanding of the main features driving predictions. To resolve the gap in standardized methodologies for PV forecasting, a structured ML evaluation framework is proposed to establish a systematic approach, which adapts with the ever-evolving ML domain.

The aim of this research is to predict system temperature and humidity in the PV environment, because these factors have a significant effect on efficiency and also the life span of a PV system. Unlike other studies involving the forecasting of weather-related variables, this study is highly applicable towards the realization of renewable energy generation forecasts of specific interest for the renewables industry, making it far more relevant to real-world concerns. Using nine different ML models, the study demonstrates a comparative methodology to examine the suitability of the different techniques for the purpose of PV optimization. Moreover, SHAP analysis integration improves model interpretability and provides essential insights into factors influencing PV performance within the environment. This case study’s finding enhance predictions for maintenance and monitoring in real-time for solar energy systems, leading to sustainable harvesting.

The study is structured systematically to ensure a clear progression from data acquisition to model evaluation. The Introduction provides background on PV systems, emphasizing the impact of environmental factors like temperature and humidity on their efficiency. It highlights the need for accurate forecasting using ML and justifies the comparative analysis of various ML models. The Methodology section details the research approach, beginning with Data Presentation, which describes the dataset, statistical summaries, and key environmental parameters. This is followed by an overview of ML Algorithms, covering model selection, training, and evaluation using MAE, RMSE, and R². The Results and Discussion section presents model comparisons, highlighting the superior performance of ensemble-based models like XGBoost and RF through visual analysis, including scatter plots, violin plots, and SHAP analysis for feature importance interpretation. The Future Work section explores advanced ML techniques, such as deep learning, optimization strategies, and real-time deployment for PV forecasting. Finally, the Conclusion summarizes key findings, emphasizing the advantage of ensemble models over traditional methods and suggesting further research to enhance real-time monitoring and predictive accuracy in PV systems.

## Methodology

### Data presentation

In this section, the important variables in the dataset are shown visually and descriptive statistics are provided. It contains environmental and sensor based data including temperature, humidity, Ultraviolet (UV) index, voltage, current and illuminance.

In this study, the 5,000 sample dataset was divided into training and testing subsets for reliable assessment of the ML models. 80% of the dataset (four thousand samples) were held out for model training and allowed models to find patterns that existed under the surface information. The remaining 20% (1,000 samples) will be reserved for testing the models’ predicted ability on unobserved data and providing an independent evaluation. This split of the data makes the model not overfit the models on the dataset and also are able to generalize the models on the data which will be provided to the models at the end and beyond the training set.

**Table 1:** Summary statistics of environmental data.

Statistic	Count	Mean	Std	Min	25%	50%	75%	Max
Temperature	5000	15.059	11.513	0.6	6.5	10.9	24.7	43.8
Humidity	5000	31.749	12.012	10.1	22.4	30.1	40.9	65.2
UV	5000	1.393	2.321	0	0	0	1.81	8.41
Voltage	5000	6.144	9.093	0	0	0	16.38	22.41
Current	5000	1.012	1.239	0.12	0.17	0.19	2.42	3.21
Illuminance	5000	484.307	460.685	46.45	52.4	56.9	1007.412	1012.3

The study’s primary variables—temperature, humidity, UV index, voltage, current, and illuminance—are summarized statistically in Table 1. With 5,000 observations for each variable in the dataset, a thorough foundation for comparing ML methods for temperature and humidity prediction in PV settings is provided.

With a standard deviation of 11.51 °C and a mean value of almost 15.06 °C, the temperature data reveals a broad range of observed temperatures. The measured temperatures range from 0.60 °C to 43.80 °C, representing the variety of environmental conditions that were recorded.

The range of humidity levels is from 10.10 to 65.20%, with a mean of 31.75% and a standard deviation of 12.01%. For the purpose of modelling meteorological conditions and how they affect PV systems, this humidity fluctuation is essential.

The UV index readings range from 0 to 8.41, with a mean of 1.39 and a standard deviation of 2.32. This suggests that the dataset encompasses intervals of both low and high UV exposure, which is crucial for comprehending how solar radiation affects humidity and temperature.

With averages of 6.14 V and 1.01 A, respectively, the voltage and current readings also show a great deal of variability, with the voltage peaking at 3.21 A and the current values reaching as high as 22.41 V. Analyzing the electrical performance of solar systems in various climatic situations requires an understanding of these variances.

With an approximate mean of 484.31 lx and a broad range from 46.45 lx to 1012.3 lx, the illumination clearly illustrates the breadth of different light conditions seen in the sample. Understanding how variations in light intensity impact temperature and humidity in PV settings depends on this variability.

All things considered, Table 1 offers a thorough synopsis of the dataset’s statistical characteristics, laying a strong basis for the next research and modelling projects. For the purpose of creating and assessing reliable ML models intended to precisely anticipate environmental conditions in solar systems, a wide range of data across all variables must be collected.

**Fig. 2:**
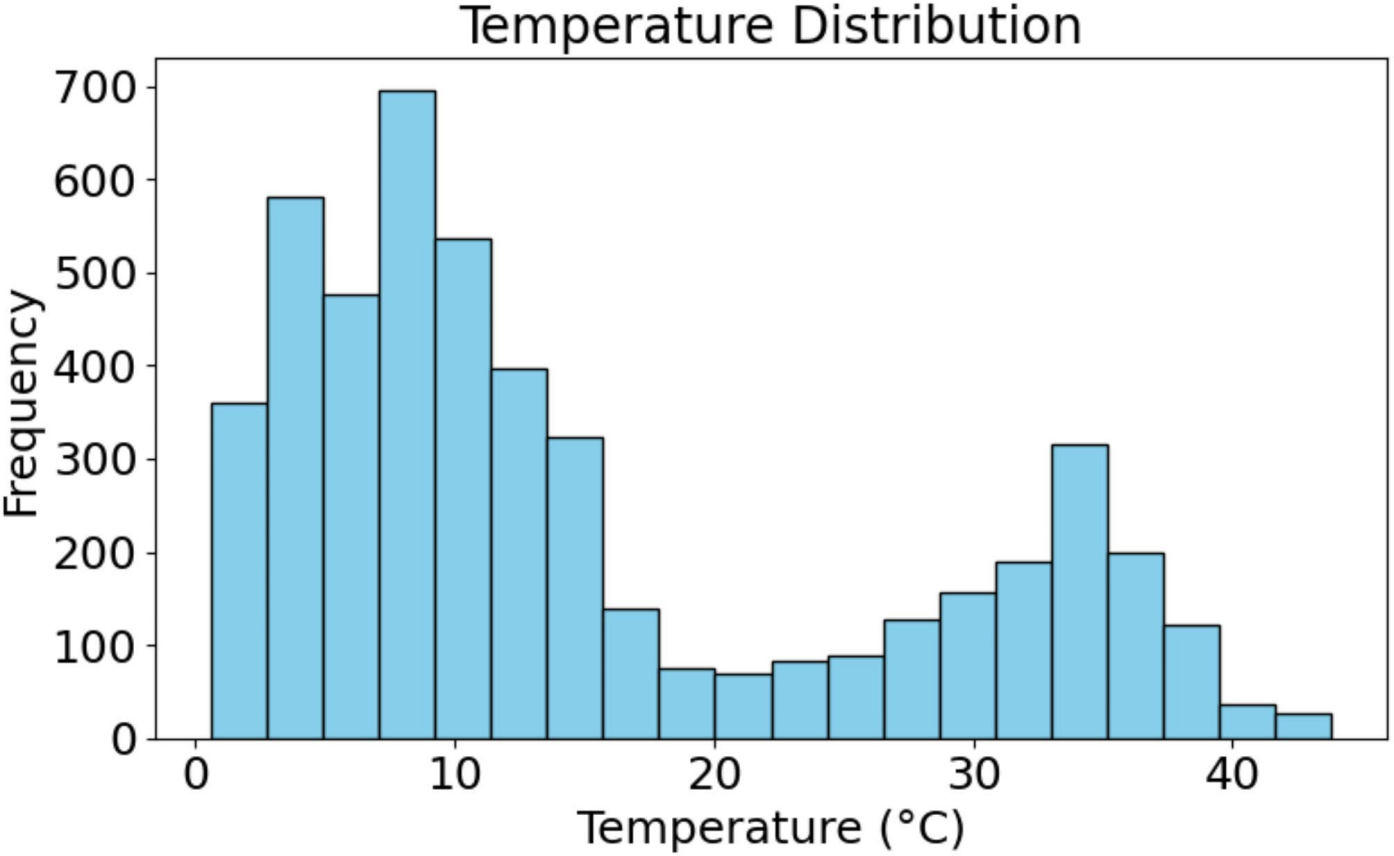
Histogram of temperature measurements: Frequency distribution of recorded temperatures in the dataset.

A range of temperature observations found in the dataset is shown in Fig. 2. Most of the temperatures recorded fall in the range 0 °C to 20 °C and thereby display a certain pattern in distribution. The most frequent reported temperatures are indicated by a prominent peak around 10 °C. This frequency declines incremental up to high temperature ranges such as from 30 °C to 40 °C, where fewer recording appeared. This means that the PV environment was predominantly characterized by lower and not very high temperatures. So, it shows not only how much heat is actually produced by this PV system but also what are particular humidity and temperature conditions in which this PV system was working, and it was working during the negative skewness of temperature distribution.

**Fig. 3:**
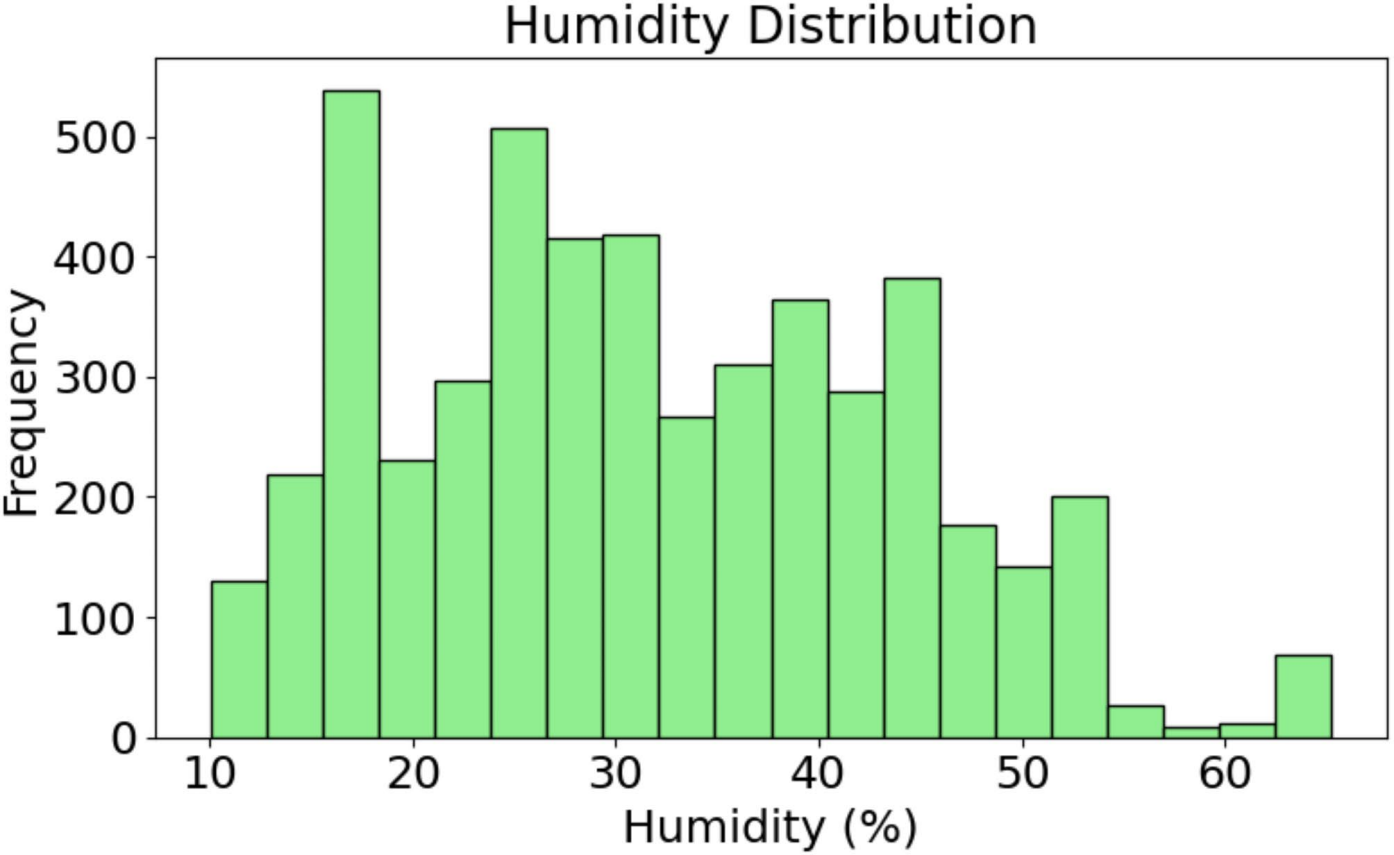
Histogram of humidity levels: Frequency distribution of recorded humidity values in the dataset.

Figure 3 displays the frequency distribution of the measured humidity values. Histogram showing a wide distribution of humidity, with the bulk of data lagging between 20% and 40%. The peak frequency occurs at 20% or so, which indicates that humidity levels in the PV environment were rather low. Above 40% it is an increasing scarce and above 60% is virtually non-existence. This distribution suggests that the PV system is regularly under dry conditions, which may be relevant in understanding how humidity influences the prediction of temperature in this type of environment.

**Fig. 4:**
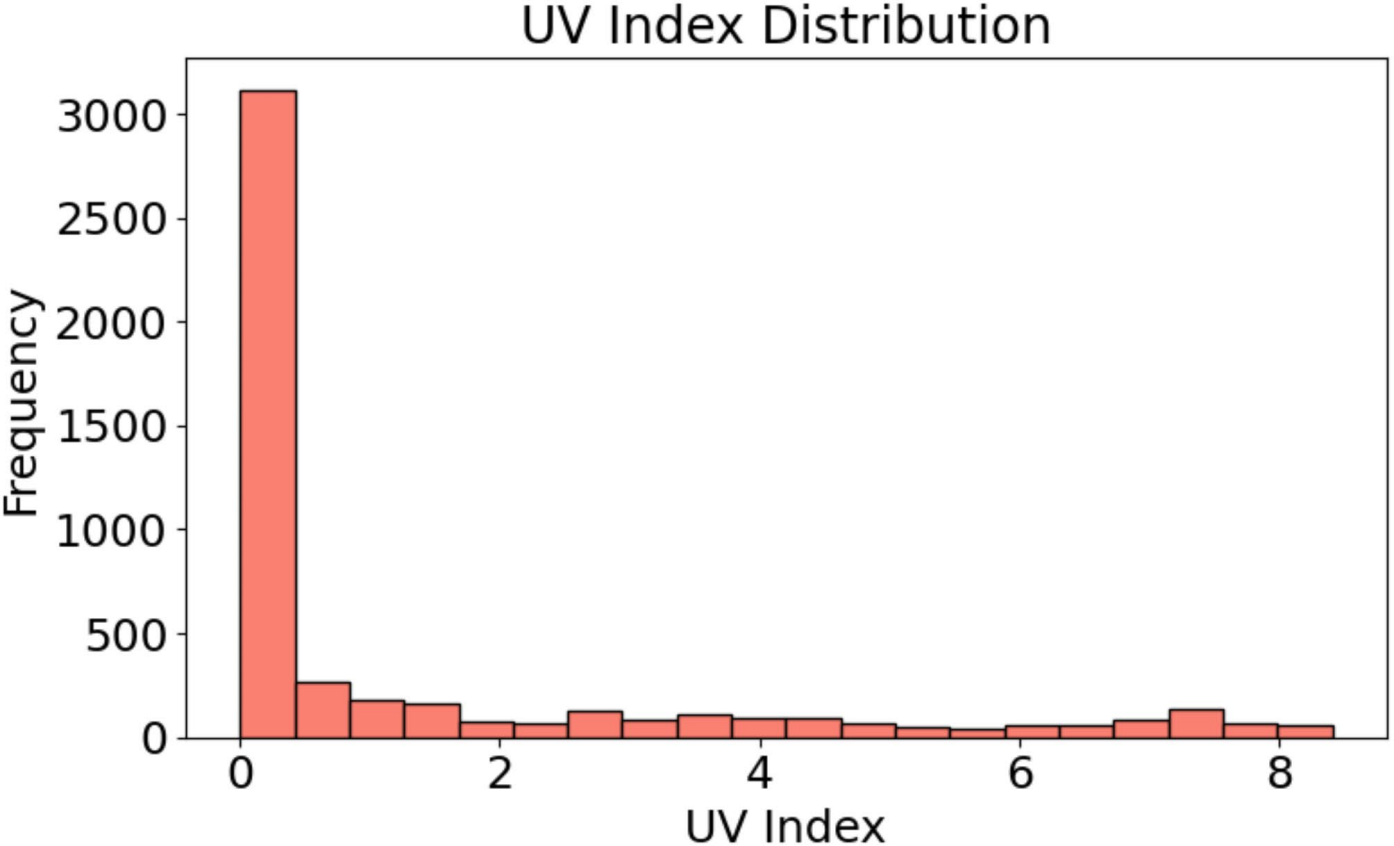
Histogram of UV index values: Frequency distribution of recorded UV index measurements in the dataset.

The distribution of UV index values is shown in Fig. 4 with most of the recorded values between 0 and 1. A prominent spike is observed at the 0 line, indicating that a significant fraction of the observations is associated with very low or negligible levels of UV exposure. With just a few quantity of data obtained beyond a UV index of 2, the frequency drastically falls as the UV index increases. The low UV index readings suggest that the PV system operates in conditions when most of the time there is minimal direct sunlight or UV exposure. Accurate projection of the effect of UV exposure on the performance and temperature conditions of the system depends on this knowledge.

**Fig. 5:**
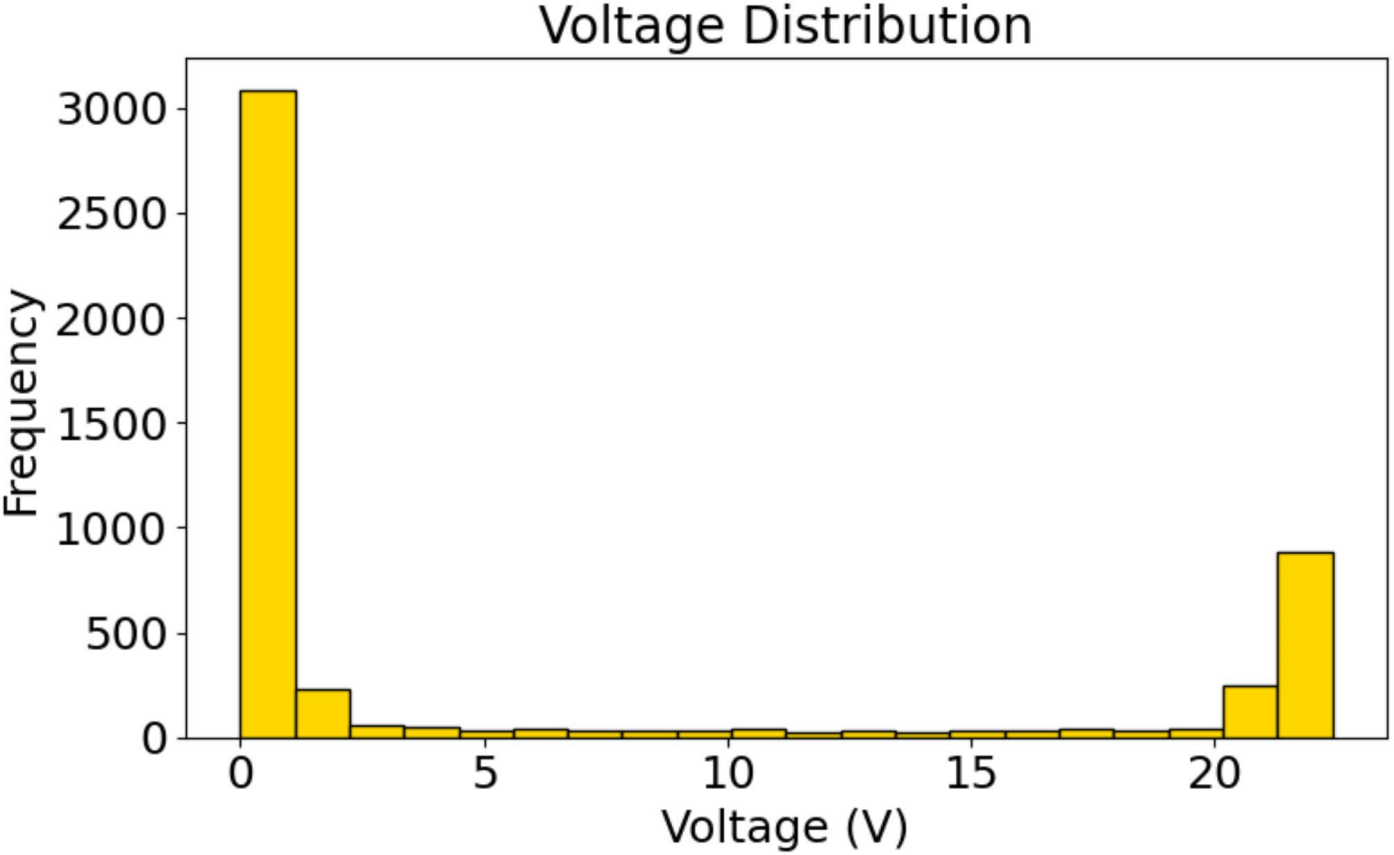
Histogram of voltage measurements: Frequency distribution of recorded voltage values in the dataset.

A bimodal pattern may be seen in the voltage measurement distribution, as seen in Fig. 5. There are a lot of readings that are concentrated around 0 V, which suggests that there are a lot of low or no voltage observations. This may be a sign of times when the PV system was not operating at all or was only getting very little electricity. The second peak appears at about 20 V, indicating that the system regularly functioned at this voltage level while it was in operation. The difference between these two peaks illustrates how the PV system operates, showing dramatic differences between periods of activity and inactivity.

**Fig. 6:**
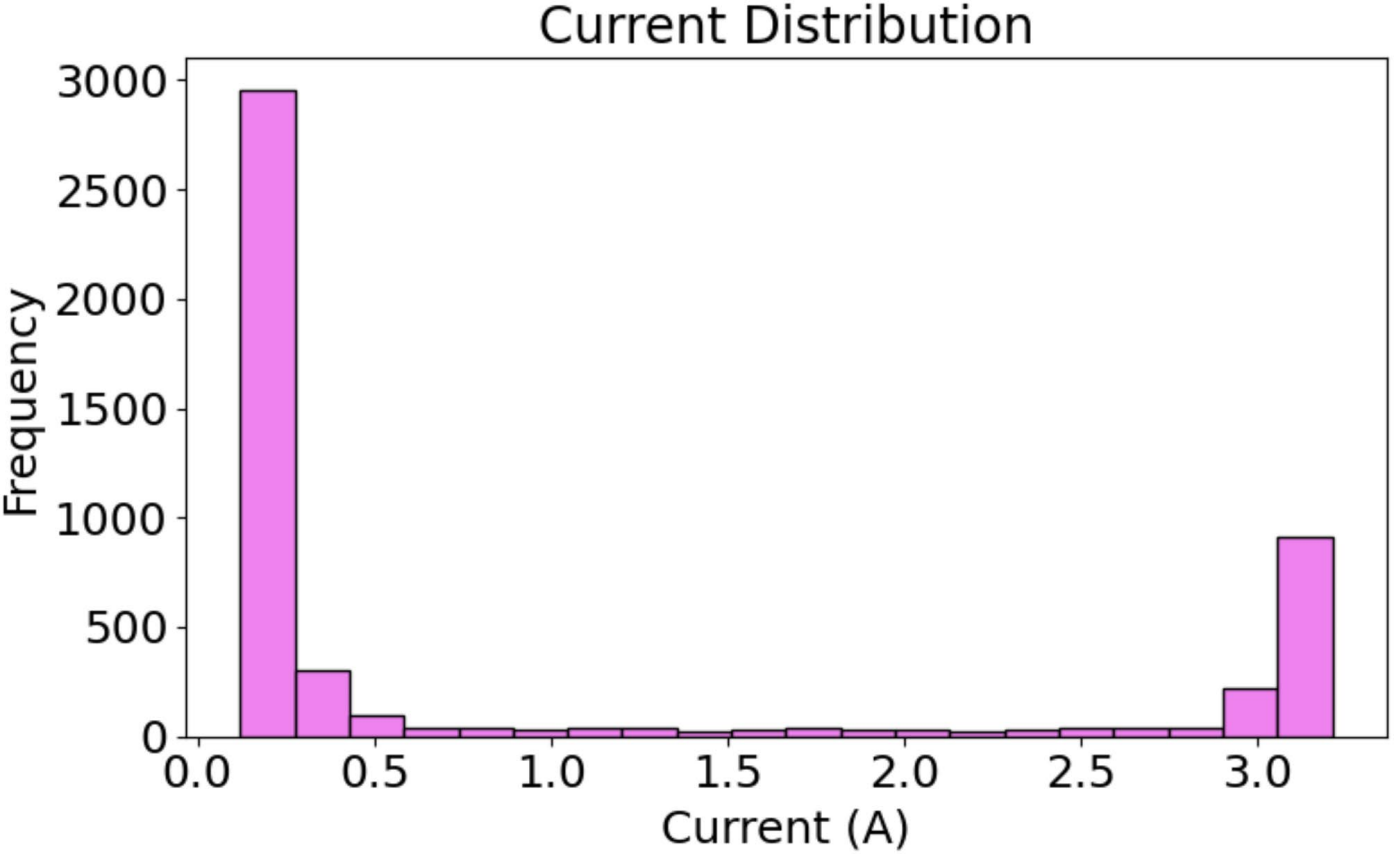
Histogram of current measurements: Frequency distribution of recorded current values in the dataset.

Figure 6 shows a distribution of current measurements, which is also bimodal, similar to the voltage distribution. The maximum peak at 0 A indicated that the system often failed to detect current during periods of idleness or poor energy output. A second, lesser peak appears at 3 A, indicating that this level was primarily where current was created. The fact that the solar system often alternates between periods of inactivity and moderate energy output is further highlighted by this bimodal pattern.

**Fig. 7:**
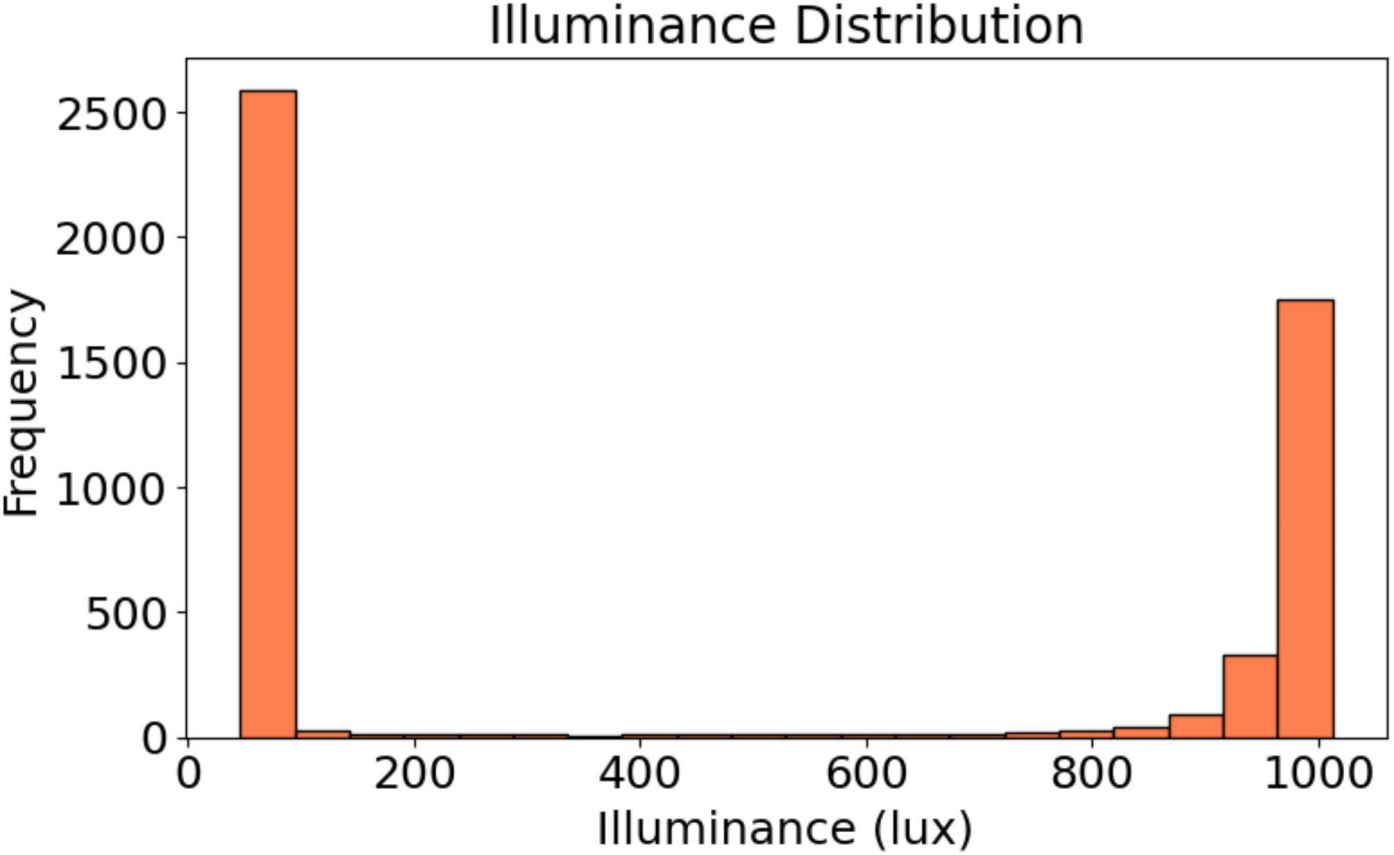
Histogram of illuminance levels: Frequency distribution of recorded illuminance values in the dataset.

Similarly, the illuminance values presented in Fig. 7 also display bimodal distribution. The large clustering of observations around 0 lx suggests that the PV system faced conditions with little or no light for a substantial fraction of the time. This could refer to snapshots taken through the night or during other low light conditions. The second peak, detected at approximately 1000 lx, shows that under light stimulation the system frequently processed at relatively high illuminance levels. The distance between these two peaks shows both an incredible low and high illuminance by the system, with only small points of data in between.

### Machine learning algorithms

**Fig. 8:**
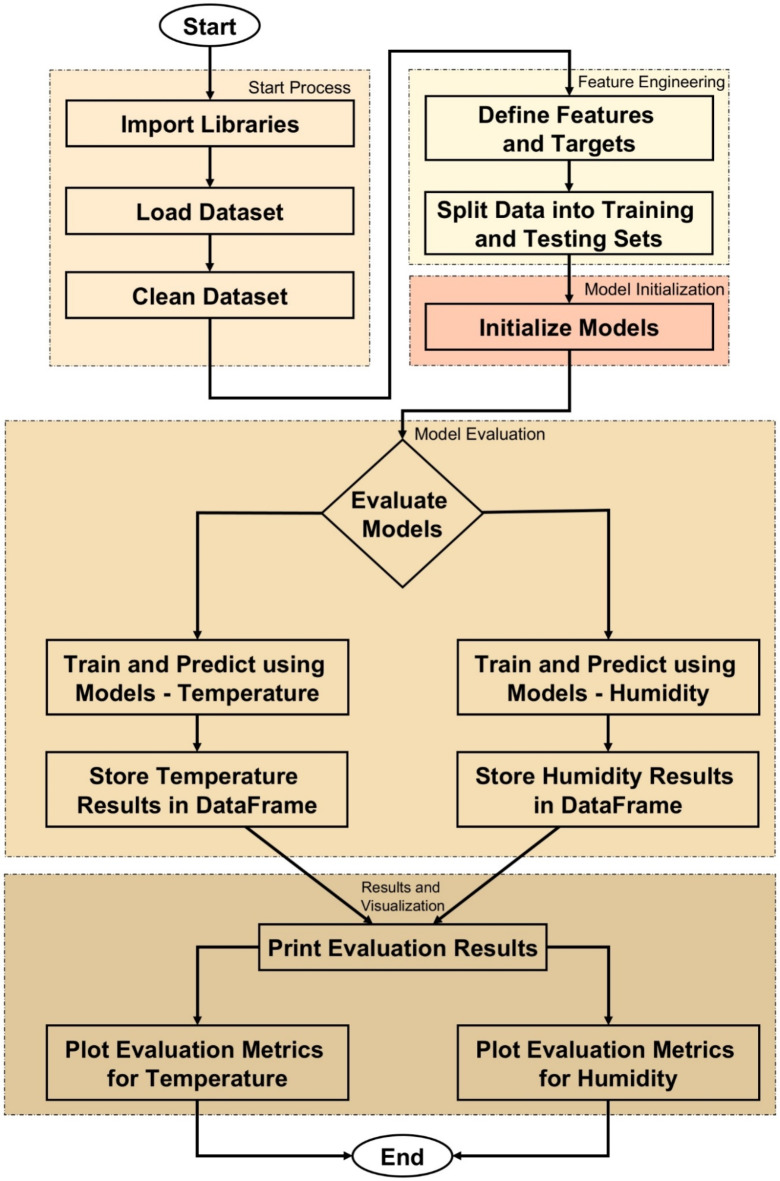
ML workflow for temperature and humidity prediction in PV environments: A step-by-step representation of data preprocessing, feature selection, model training, evaluation, and visualization in the predictive modeling process.

ML Algorithms section provides a detailed examination of ML methods applied for the temperature and humidity prediction in solar systems. In this section, the model selection, training, and assessment procedures are examined, and the pros and cons of each approach are discussed. The analysis, incorporating factors such as complexity, feature handling, and interpretability, allows understanding of the trade-offs between model performance and usability. An analysis of ML techniques used in this study is also presented in this section, including a comparison framework to understand the efficacy of each model using Fig. 8; Table 2.

Through their capacity to manage substantial datasets and uncover intricate correlations among variables, ML models have been widely utilized in a range of environmental engineering fields. Other than predictive modeling monitoring in PV-based surroundings, ML techniques such as Artificial Neural Networks (ANN), Support Vector Machines (SVM), and ensemble-based of more than a few techniques are able to doing well in equivalent fields such as hydrological forecasting and water quality assessment. As an example, ANN and SVM were used successfully for river classification and to improve water quality monitoring in monsoonal environments, showing the adaptability of the algorithms for environmental modeling^23^. Similarly, hybrid ML approaches combining ANN, Adaptive Neuro-Fuzzy Inference System (ANFIS), and Multiple Linear Regression (MLR) have been applied to predict heavy metal adsorption in water treatment, demonstrating their capability in handling nonlinear relationships and multi-variable dependencies^24^. Furthermore, ensemble learning methods such as RF and optimized SVM models have been used for water quality index prediction, emphasizing the benefits of feature selection and model tuning for improving predictive accuracy^25^. These studies reinforce the versatility of ML models in data-driven environmental predictions, supporting their applicability to temperature and humidity forecasting in PV systems.

The whole ML pipeline for predicting temperature and humidity in PV situations is depicted in Fig. 8. Importing the required libraries—such as pandas, numpy, and many ML and visualization libraries—is the first step in the process. The dataset is then entered into the software, where it is cleaned up by removing superfluous columns and using the median to manage missing values. This ensures that the data is ready for training models.

The flowchart then moves on to the feature engineering stage, when the target variables—heat and humidity—are separated from the pertinent characteristics, or independent variables. After that, the dataset is divided into training and testing sets so that the models may be trained on some data and their performance can be assessed on the remaining data.

Several ML models are initialised after feature engineering. These models include more sophisticated models like RF, GB, AdaBoost, and XGBoost in addition to more conventional models like DT and LR.

After that, the flowchart splits into two independent processes: one for predicting temperature and the other for predicting humidity. Predictions are made once the models in each branch have been trained on the appropriate data. Important measures, such as the R^2^, RMSE, and MAE, are used to assess each model’s performance. To make it easier to compare the models, these assessment results are kept in DataFrames.

Lastly, bar charts that provide the performance metrics for the temperature and humidity forecasts are presented along with the data. When the assessment and visualization process is finished, the flowchart ends, clearly illustrating how well each model performs in forecasting temperature and humidity in PV situations.

A thorough comparison of ML techniques used in PV settings to forecast temperature and humidity is presented in Table 2. An understanding of the advantages and disadvantages of each of these algorithms is provided in Table 2, which lists the salient features of each algorithm.

The basic strategy used by every model is the kind of algorithm. For example, RR, Lasso Regression and LR are basic linear models that assume linear relationship between features and target variables^13^. These models are often the first to be considered when simplicity and interpretability are important. However, to deal with non-linear relationships and interactions between features, more advanced ensemble methods such as RF, AdaBoost, GB and XGBoost are designed^26^. Consequently, these methods tend to achieve higher predictive accuracy, but at the cost of increased computational complexity and lower interpretability.

Each model is incredibly different in terms of complexity. Linear models are suitable in cases in which computational resources are scarce because they are simple to use and computationally economical^13^. However, models such as XGBoost and GB take more time and processing resource owing to their repeated training of many weak learners (typically DTs), and aggregation of predictions from them to improve the accuracy^17^.

Feature management varies in its sensitivity to data preparation when used with the models. For instance, SVR and linear models are sensitive to the size of the features, and need to be normalized or standardized for best performance^19^. However, ensemble techniques like RF and XGBoost are both capable of handling numerical and categorical data and are more impervious to feature scale changes^17^. Lasso Regression shines due to its ability to perform automated feature selection by efficiently reducing model complexity and dealing with multicollinearity issues^13^. This is achieved by shrinking the coefficients of less relevant features to zero.

Another crucial component to take into account is interpretability, particularly in applications where it’s crucial to comprehend the model’s decision-making procedure. Since the model coefficients clearly show how each feature and the target variable relate to one another, RR and LR are very interpretable^13^. Through their visual depiction, DT models also provide great interpretability, providing obvious insights into the decision-making process at each split in the tree. Nevertheless, interpretability declines with the use of more sophisticated models such as GB, AdaBoost, and XGBoost^26^. Because it is challenging to determine how certain variables affect the final prediction due to the ensemble of weak learners, these models are sometimes referred to as “black box” models. In spite of this, they often perform better in terms of predicted accuracy than simpler models, particularly when working with complicated datasets.

An overall analysis of trade-offs between various algorithms, such as SVR, Lasso Regression, RR, LR, AdaBoost, GB, DT, RF, and XGBoost, based on complexity, feature handling, and interpretability is presented in Table 2^27–29^. Simpler models can be more transparent, and require less processing power, but capture less intricate patterns in the data, whereas more complicated ensemble approaches capture more intricate patterns in the data at the expense of interpretability. Comparing these models is necessary to understand for what type of temperature and humidity prediction tasks each model is applicable, taking into account the accuracy versus interpretability trade off specific to the application.

**Table 2:** Comparative analysis of ML algorithms used for temperature and humidity prediction.

Algorithm	Type	Complexity	Feature handling	Interpretability
SVR	Support vector machine	High	Requires normalization, sensitive to feature scaling	Low – Black box model, difficult to interpret
Lasso regression	LR with Lasso regularization (L1)	Medium	Automatically performs feature selection (sparse coefficients)	Medium – Coefficients can be interpreted
RR	LR with ridge regularization (L2)	Medium	Handles multicollinearity, requires feature scaling	Medium – Coefficients can be interpreted
LR	LR	Low	Sensitive to multicollinearity, requires feature scaling	High – Simple and easily interpretable
AdaBoost	Ensemble method (Boosting)	High	Handles a variety of features, may require pre-processing	Low – Complex ensemble of weak learners
GB	Ensemble method (Boosting)	High	Handles a variety of features, robust to feature scaling	Low – Complex ensemble, difficult to interpret
DT	DT	Medium	Handles categorical and numerical features, no scaling needed	High – Easily interpretable with visual trees
RF	Ensemble method (Bagging)	High	Handles a variety of features, robust to scaling	Medium – Individual trees are interpretable, but the forest is less so
XGBoost	Ensemble method (Boosting)	High	Handles a variety of features, requires careful tuning	Low – Highly complex, difficult to interpret

This study did not utilize any decomposition strategy to address and reduce data noise prior to training the ML models. The main reason for this choice was to evaluate the inherent forecasting skills of various ML models in managing real-world environmental data without any preprocessing. High-frequency noise in temperature and humidity data can be filtered using decomposition techniques as Wavelet Transform, Empirical Mode Decomposition (EMD), or Seasonal-Trend Decomposition (STL), therefore adjusting the temperature and humidity data. Having said that, because they may find trends among several decision trees, some ensemble techniques like RF and XGBoost are naturally resilient to noise. On the other hand, simpler models like LR and SVR may have been more vulnerable to noise and they made more mistakes. Decomposition was not performed so some of this may have contributed to poor prediction, especially if the models can not handle noise. Future research will focus on the addition of decomposition methods as part of the ensemble model to understand their effect on the prediction accuracy and for generating more reliable predictions in the PV environment.

The study utilized nine different regression models from several libraries, including classical methods such as Decision Tree and LR, and ensemble methods such as RF, GB, and XGBoost, as summarized in Table 3. This study used their respective Python classes to access each model (for instance, “sklearn.tree.DecisionTreeRegressor” for the Decision Tree). By accommodating this suite of algorithms, a general comparison of predictive power and computational complexity over the PV setting can be performed.

**Table 3:** Model and library/class.

Model	Library/Class
Decision tree	“sklearn.tree.DecisionTreeRegressor”
LR	“sklearn.linear_model.LinearRegression”
RR	“sklearn.linear_model.Ridge”
Lasso regression	“sklearn.linear_model.Lasso”
SVR	“sklearn.svm.SVR”
RF	“sklearn.ensemble.RandomForestRegressor”
GB	“sklearn.ensemble.GradientBoostingRegressor”
AdaBoost	“sklearn.ensemble.AdaBoostRegressor”
XGBoost	“xgboost.XGBRegressor”

As illustrated in Table 4, each regression model relies on a distinct set of key hyperparameters that regulate its training process and overall performance. Simpler models such as LR and RR have fewer settings (for example., “fit_intercept” or “alpha”), whereas more complex algorithms like XGBoost feature numerous adjustable parameters, including “learning_rate,” “max_depth,” and “subsample.” This variety highlights how tuning specific hyperparameters can significantly influence model behavior in terms of both predictive accuracy and computational complexity.

**Table 4:** Model and key hyperparameters.

Model	Key hyperparameters
Decision tree	“random_state”, “max_depth”, “min_samples_split”, “min_samples_leaf”
LR	“fit_intercept”, “normalize” (deprecated in newer versions)
RR	“alpha”, “fit_intercept”
Lasso Regression	“alpha”, “fit_intercept”
SVR	“kernel”, “C” (regularization), “epsilon” (ε-insensitive), “gamma” (for RBF kernel)
RF	“random_state”, “n_estimators”, “max_depth”
GB	“random_state”, “n_estimators”, “learning_rate”, “max_depth”
AdaBoost	“random_state”, “n_estimators”, “learning_rate”
XGBoost	“random_state”, “n_estimators”, “learning_rate”, “max_depth”, “subsample”, “colsample_bytree”

As highlighted in Table 5, each model was initially configured with default parameter settings to enable a fair, “out of the box” comparison. These defaults—which range from disabling maximum depth in Decision Trees (“max_depth = None”) to using moderate ensemble sizes (e.g., “n_estimators = 100” for both RF and GB)—generally offer balanced performance without extensive tuning. The accompanying remarks in Table 5 clarify how these defaults influence each algorithm’s behavior, underscoring, for example, the sensitivity of SVR to “C,” “epsilon,” and “gamma,” or the risk of overfitting in boosting-based models with large “n_estimators.”

**Table 5:** Default values used and remarks.

Model	Default values used	Remarks
Decision tree	“random_state = 42”, “max_depth = None”, “min_samples_split = 2”, “min_samples_leaf = 1”	No maximum depth (splits until leaves are pure). Larger depths risk overfitting; smaller depths can underfit.
LR	“fit_intercept = True”, “normalize = False” (deprecated, default behavior)	Assumes a linear relationship; no direct regularization. Sensitive to multicollinearity and outliers.
RR	“alpha = 1.0”, “fit_intercept = True”	L2 regularization shrinks coefficients; helps with multicollinearity and reduces overfitting.
Lasso regression	“alpha = 1.0”, “fit_intercept = True”	L1 regularization encourages sparsity (coefficient = 0) for less important features.
SVR	“kernel=’rbf’”, “C = 1.0”, “epsilon = 0.1”, “gamma=’scale’”	Learns a function within an ε-tube. Sensitive to “C”, “epsilon”, and “gamma”; may require careful scaling and tuning for best results.
RF	“random_state = 42”, “n_estimators = 100”, “max_depth = None”	Ensemble of decision trees via bagging. Generally robust to outliers and can handle high-dimensional data.
GB	“random_state = 42”, “n_estimators = 100”, “learning_rate = 0.1”, “max_depth = 3”	Sequentially adds weak learners to minimize loss. Can overfit if “n_estimators” is large without regularization.
AdaBoost	“random_state = 42”, “n_estimators = 50”, “learning_rate = 1.0”	Boosts performance by focusing on mis-predicted samples. Works well with shallow base estimators (e.g., short decision trees).
XGBoost	“random_state = 42”, “n_estimators = 100”, “learning_rate = 0.1”, “max_depth = 6”, “subsample = 1.0”, “colsample_bytree = 1.0”	Efficient gradient boosting library with built-in regularization and tree-pruning. Can overfit if parameters are not tuned.

### Evaluation metrics

This study included three primary assessment measures to gauge the effectiveness of ML models in predicting temperature and humidity in PV settings. The metrics MAE, RMSE, and R^2^ provide a thorough assessment of the accuracy and goodness-of-fit of the models^30–33^. The MAE is a metric used to quantify the average size of mistakes in a given collection of predictions, regardless of their direction. The calculation determines the absolute discrepancy between the anticipated values and the actual values, providing a readily understandable measure of the accuracy of the forecast. Smaller MAE values imply superior model performance. Equation (1) is the formula for MAE. The RMSE is a measure that calculates the square root of the average of the squared discrepancies between projected values and actual values. RMSE is more sensitive to outliers compared to MAE since it penalizes greater mistakes more heavily by squaring the residuals. Similar to MAE, lower RMSE readings imply higher levels of prediction accuracy. RMSE is a valuable tool for comprehending the scale of prediction mistakes in the same units as the output variable. Equation (2) provides the formula for calculating the RMSE.

R^2^ quantifies the amount of the variation in the dependent variable that can be accurately predicted by the independent variables. The metric offers a measure of how well the model fits the data, with values closer to 1 indicating a higher level of fit. A value of 1 for the R^2^ shows that the model completely accounts for the variation in the data, while a value of 0 indicates that the model does not account for any of the variation. Equation (3) displays the formula R^2^.

1 $$\:MAE=\frac{1}{n}{\sum\:}_{i=1}^{n}\left|{y}_{i}-{\widehat{y}}_{i}\right|$$

2 $$\:RMSE=\sqrt{\frac{1}{n}{\sum\:}_{i=1}^{n}{\left({y}_{i}-{\widehat{y}}_{i}\right)}^{2}}$$

3 $$\:{R}^{2}=1-\frac{{\sum\:}_{i=1}^{n}{({y}_{i}-{\widehat{y}}_{i})}^{2}}{{\sum\:}_{i=1}^{n}{({y}_{i}-\stackrel{̄}{y})}^{2}}$$

Recent studies in hydrology and climate forecasting have introduced alternative performance indices, such as the Combined Accuracy (CA) index, which integrates multiple error measures into a single metric to improve model assessment^34,35^. The CA index has been applied in streamflow and hydroclimatic forecasting to enhance interpretability and provide a holistic evaluation of predictive performance. Future studies could explore the applicability of the CA index in ML-based temperature and humidity prediction models to determine whether it offers advantages over conventional accuracy measures.

## Results and discussion

### Temperature prediction results

This subsection presents the performance evaluation of different ML models for temperature prediction using MAE, RMSE, and R² metrics. The results highlight the strengths and weaknesses of each model in capturing temperature variations in PV environments.

In this section, the study uses three important metrics—MAE, RMSE, and R^2^—to assess the effectiveness of several ML models for temperature prediction. Table 6 shows rounded to the closest thousandth the performance evaluation results for temperature prediction for every model across these criteria. Table 6 ranks the models from least to most efficient using the R^2^ measure.

**Fig. 9:**
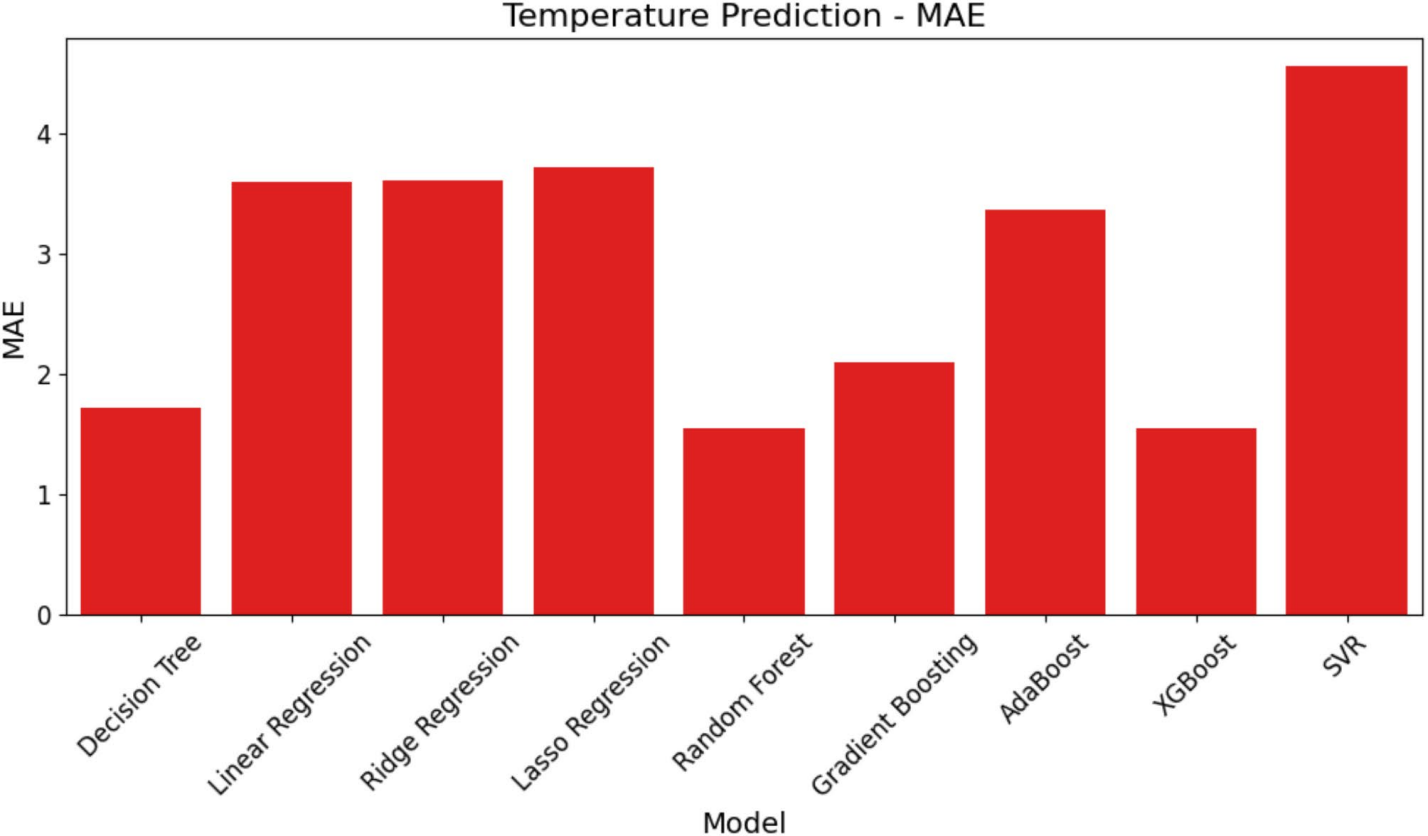
MAE comparison of ML models for temperature prediction.

**Fig. 10:**
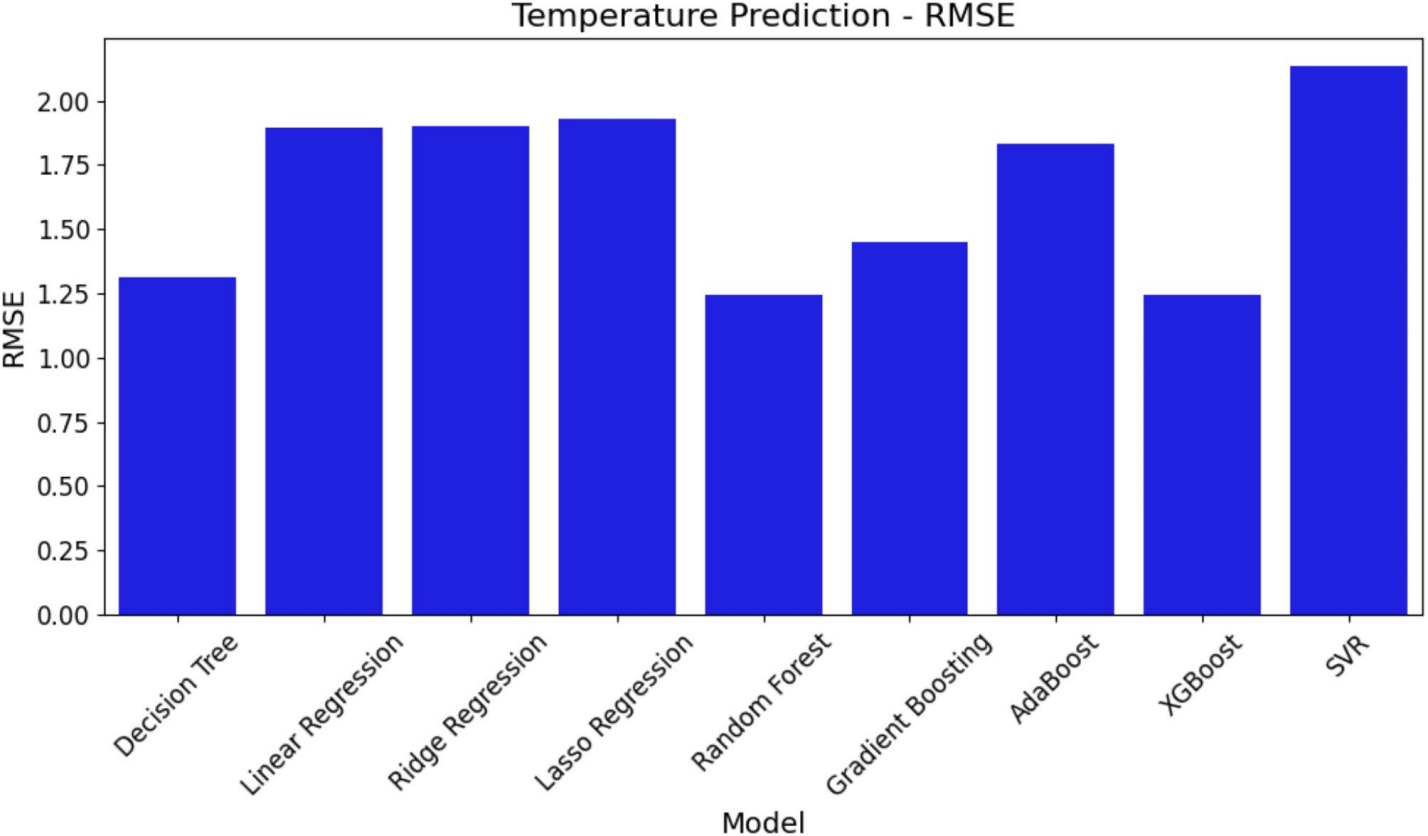
RMSE comparison of ML models for temperature prediction.

**Fig. 11:**
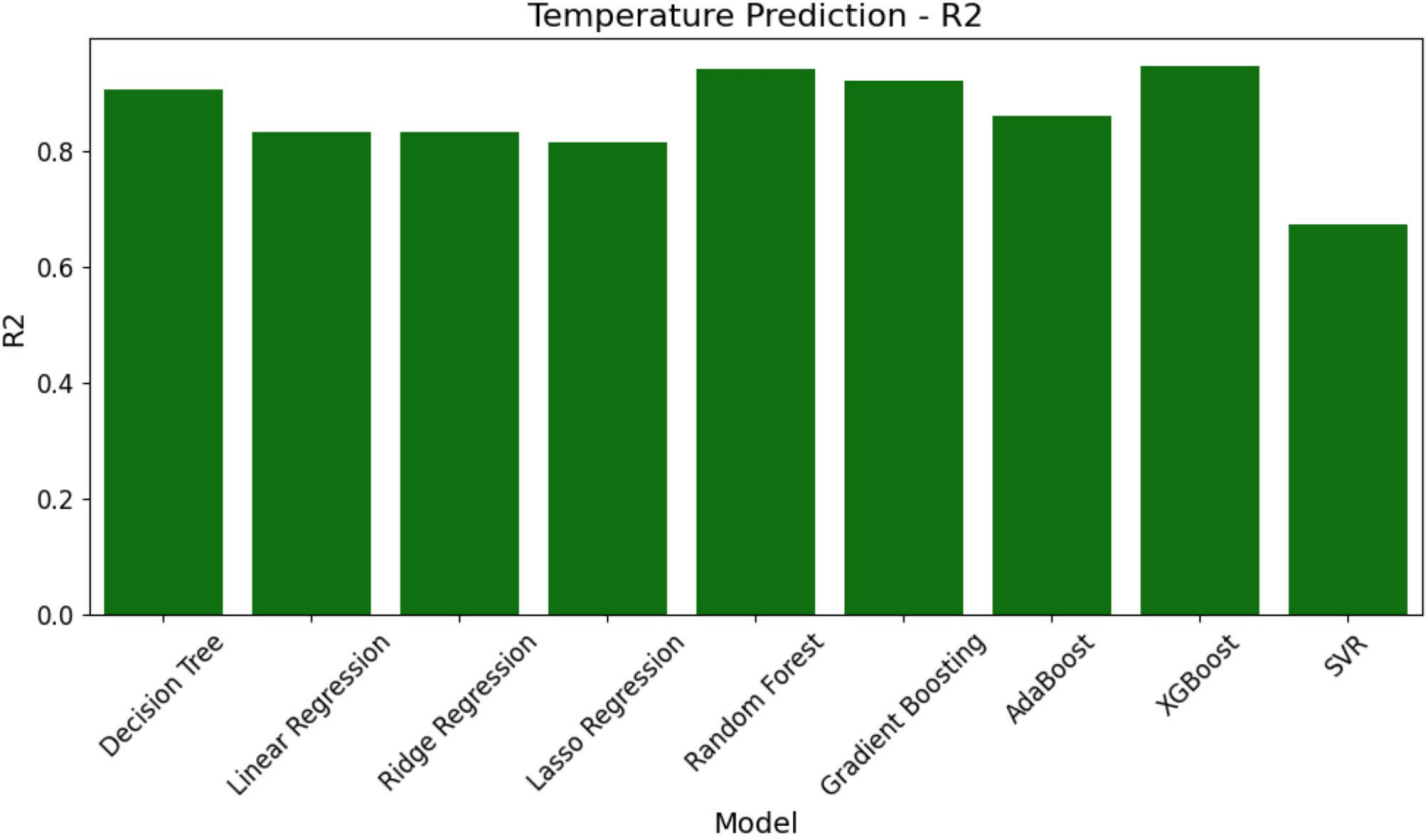
R^2^ comparison of ML models for temperature prediction.

Figures 9, 10 and  11 exhibit a graphic depiction of the model performance concerning MAE, RMSE, and R^2^ values. These graphs offer a simple reading of the errors and expected accuracy of the many models.

SVR has the highest MAE, suggesting the largest average divergence from the actual temperature readings, as shown in Fig. 9, which shows the MAE for each model. XGBoost and RF, on the other hand, display significantly lower MAE values, indicating improved temperature forecast accuracy.

Figure 10 presents the RMSE values for each model, which reflect the square root of the average squared errors. Similar to the MAE, SVR demonstrates the highest RMSE, while XGBoost and RF again stand out with the lowest RMSE values, reinforcing their strong predictive performance.

The R^2^ values quantifying the amount of variability as the model accounts for in the temperature data are presented in Fig. 11. The higher R^2^ values represent a better fit of the model. RF and XGBoost have the highest R^2^ value, while SVR lags behind with the least R^2^ value, pointing out it fits worse than the other models.

This together with the data in Table 6 shows that XGBoost and RF are most efficient for temperature prediction because they have the lowest errors and the best fit to the data, whereas SVR is the worst across all metrics of evaluation.

**Table 6:** Performance evaluation of ML models for temperature prediction.

Model	MAE	RMSE	R^2^
SVR	4.558	2.135	0.674
Lasso regression	3.718	1.928	0.814
RR	3.612	1.900	0.832
LR	3.596	1.896	0.833
AdaBoost	3.368	1.835	0.860
GB	2.102	1.450	0.922
DT	1.723	1.313	0.906
RF	1.549	1.244	0.941
XGBoost	1.544	1.242	0.947

**Fig. 12:**
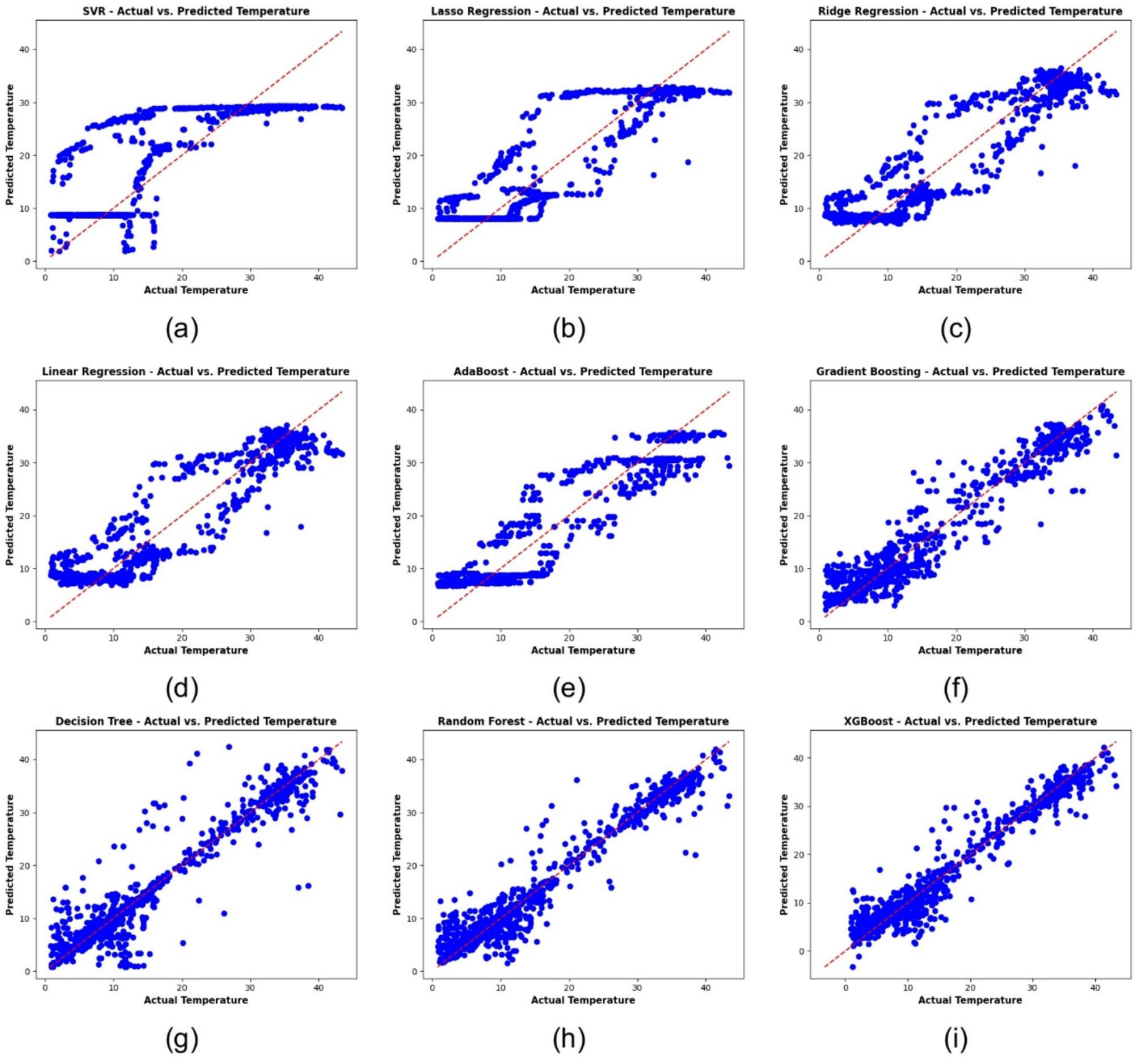
Scatter plots of [redicted vs. actual temperature across various regression models: (**a**) SVR, (**b**) Lasso regression, (**c**) RR, (**d**) LR, (**e**) AdaBoost, (**f**) GB, (**g**) Decision tree, (**h**) RF, (**i**) XGBoost.

In Fig. 12, this study presents scatter plots of predicted versus actual temperature values across nine different regression models: Fig. 12a SVR, Fig. 12b Lasso Regression, Fig. 12c RR, Fig. 12d LR, Fig. 12e AdaBoost, Fig. 12f GB, Fig. 12g Decision Tree, Fig. 12h RF, and Fig. 12i XGBoost. Points closer to the diagonal line indicate more accurate predictions, and each panel describes the correspondence between the model’s predictions and the actual temperatures. These subplots tell us at a glance which models do a better job of representing observed temperature trends.

**Fig. 13:**
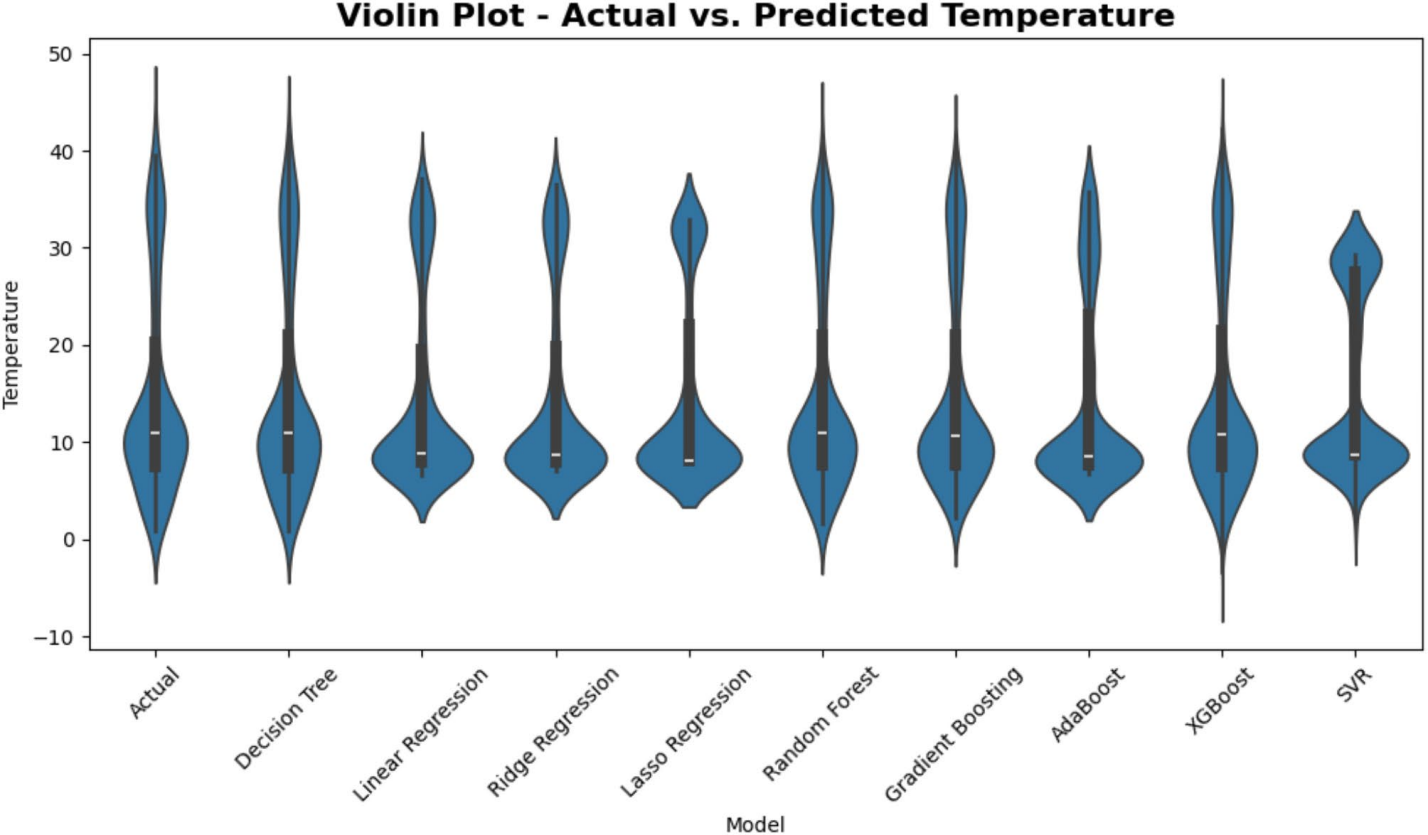
Violin plot comparison of Actual vs. Predicted temperature across ML models.

Figure 13 compares actual temperature readings with those expected by a range of regression models using violin graphs. Every “violin” shows the whole probability density of temperature values, therefore enabling the visualization of not only where expected values cluster but also the fluctuations of these values. One may get understanding of how precisely (and consistently) each model represents the observed temperature range by comparing the breadth and form of each model’s violin to the distribution of the real data.

### Humidity prediction results

This subsection analyzes the effectiveness of ML models in predicting humidity levels. The comparison based on MAE, RMSE, and R² values provides insights into each model’s ability to handle humidity fluctuations in PV environments.

Table 7 shows the ability of several ML models to predict humidity. The R^2^ values are a crucial measure of model efficiency and the study provides a ranking of the models according to this metric in the Table 7. Lower R^2^ values mean lower predictive ability, higher values mean better performance.

The results show that XGBoost performs the best with the lowest MAE (3.550), lowest RMSE (1.884) and highest R^2^ (0.744). The results of these findings indicate that XGBoost is the most accurate and reliable model to predict humidity. RF is not far behind with similar performance, having a slightly higher MAE (3.583) and RMSE (1.893) but still quite robust R^2^ (0.717). Clearly, these models are capable of successfully handling the complexity of the humidity prediction problem with a high degree of accuracy. However, SVR performs the worst with the highest MAE (6.780), the highest RMSE (2.604) and the lowest R^2^ (0.253). It appears that SSVR has difficulty capturing the fundamental patterns in the data so that the prediction is less accurate. Furthermore, other models such as Lasso Regression, RR, and LR have moderate performance with R^2^ between 0.457 and 0.468. However, when compared to XGBoost and RF, these models have much higher error metrics. AdaBoost shows a slight improvement with an R^2^ value of 0.595, but remains below the most successful models in terms of both MAE and RMSE. The DT model has acceptable performance with MAE of 3.898 and RMSE of 1.974. However, with an R^2^ of 0.649, its ability to explain variability in the humidity data is less than the more sophisticated ensemble approaches.

**Fig. 14:**
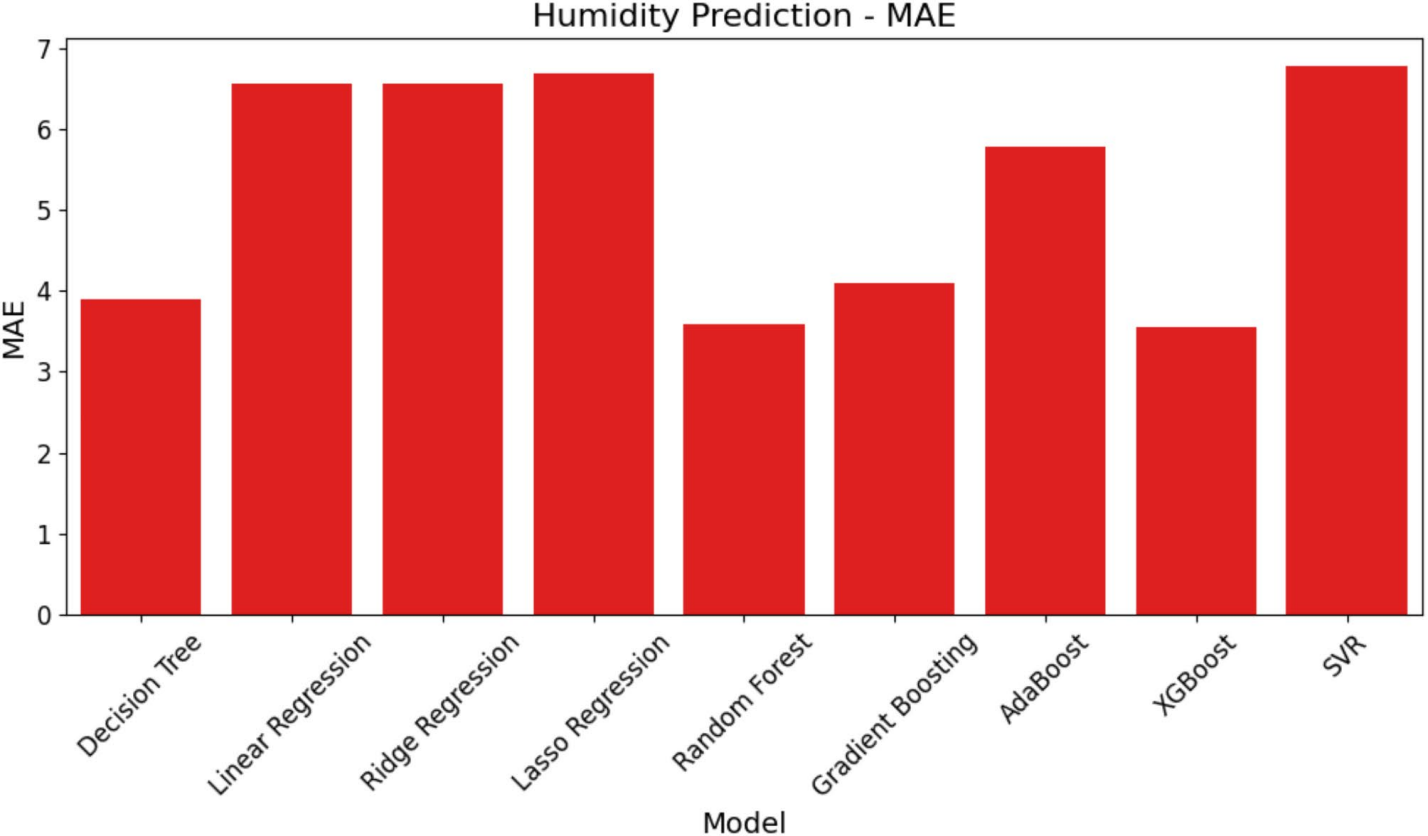
MAE comparison of ML models for humidity prediction.

Figure 14 offers a graphic representation of the models’ MAE behavior. The SVR model clearly shows with the greatest MAE that it produces the largest average inaccuracy in humidity level prediction. Conversely, RF and XGBoost have the lowest MAE values, meaning their forecasts are more accurate than those of the other models.

**Fig. 15:**
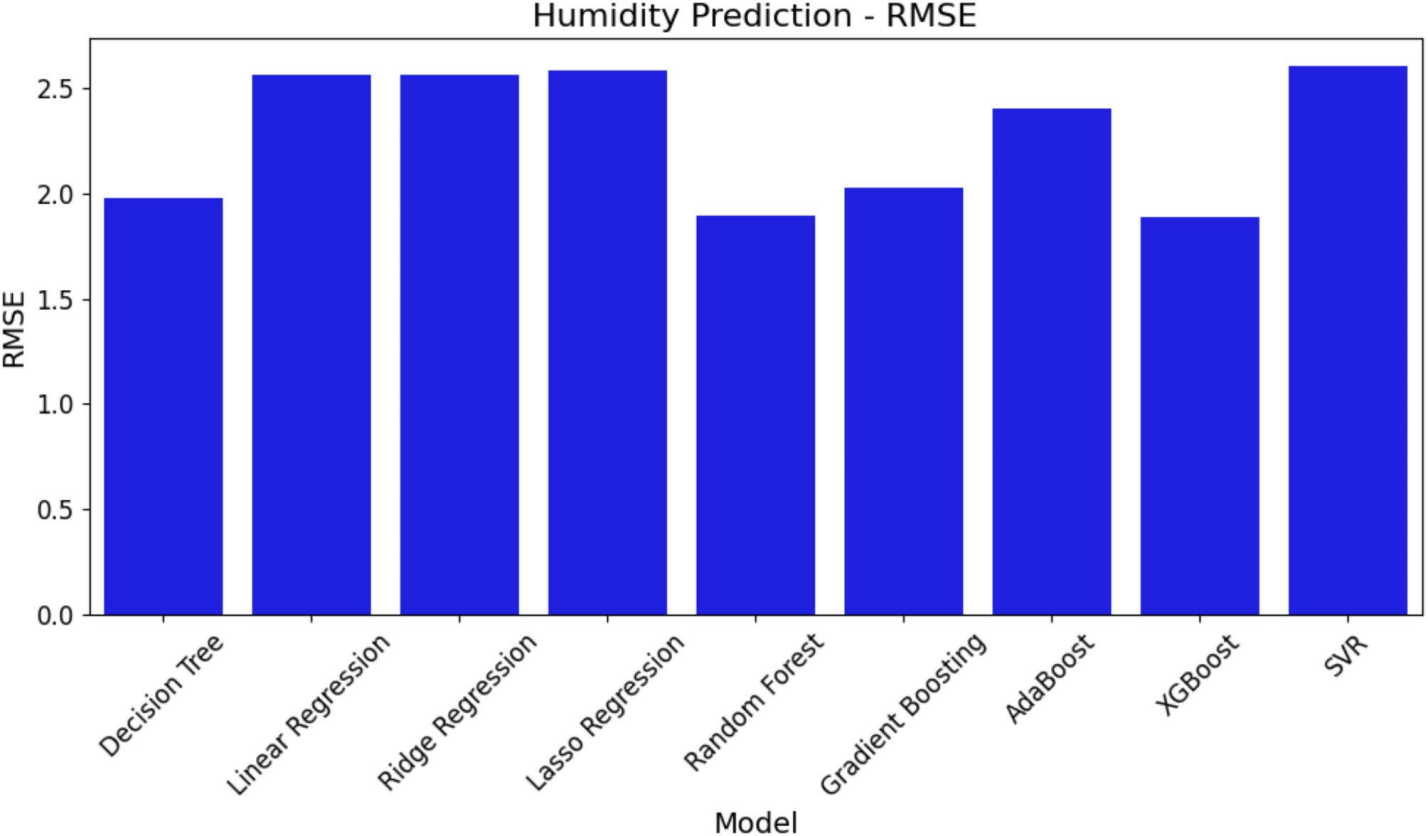
RMSE comparison of ML models for humidity prediction.

Figure 15 shows the RMSE values for every model, thereby clarifying the scale of the prediction errors. In keeping with the MAE findings, SVR shows the best RMSE—that is, equating to more prediction errors. Conversely, XGBoost and RF show improved performance once again owing to their reduced RMSE values, which results in less important error in their predictions.

**Fig. 16:**
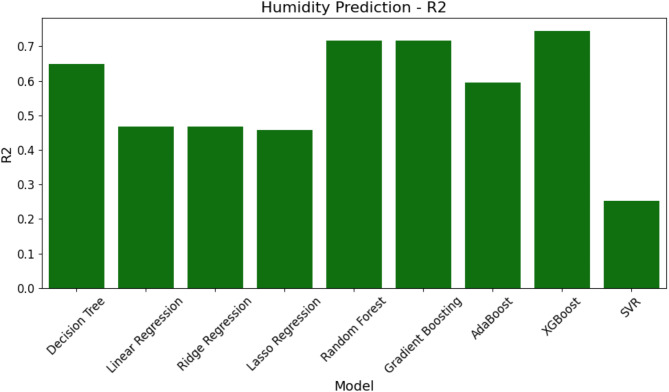
R^2^ comparison of ML models for humidity prediction.

Lastly, the models’ R^2^ values are shown in Fig. 16. The most effective model for predicting humidity is XGBoost, which has the greatest R^2^ value of 0.744. RF is next best, with an R^2^ of 0.717. SVR, on the other hand, has the lowest R^2^, highlighting even more how much less accurate it can predict than the other models.

Figures 14, 15 and 16; Table 7 show that SVR performs worse than the other models in every performance metric, while XGBoost and RF perform better at forecasting humidity and provide more accurate and consistent forecasts.

**Table 7:** Performance evaluation of ML models for humidity prediction.

Model	MAE	RMSE	*R*²
SVR	6.780	2.604	0.253
Lasso regression	6.681	2.585	0.457
RR	6.556	2.560	0.468
LR	6.555	2.560	0.468
AdaBoost	5.783	2.405	0.595
DT	3.898	1.974	0.649
GB	4.095	2.024	0.716
RF	3.583	1.893	0.717
XGBoost	3.550	1.884	0.744

**Fig. 17:**
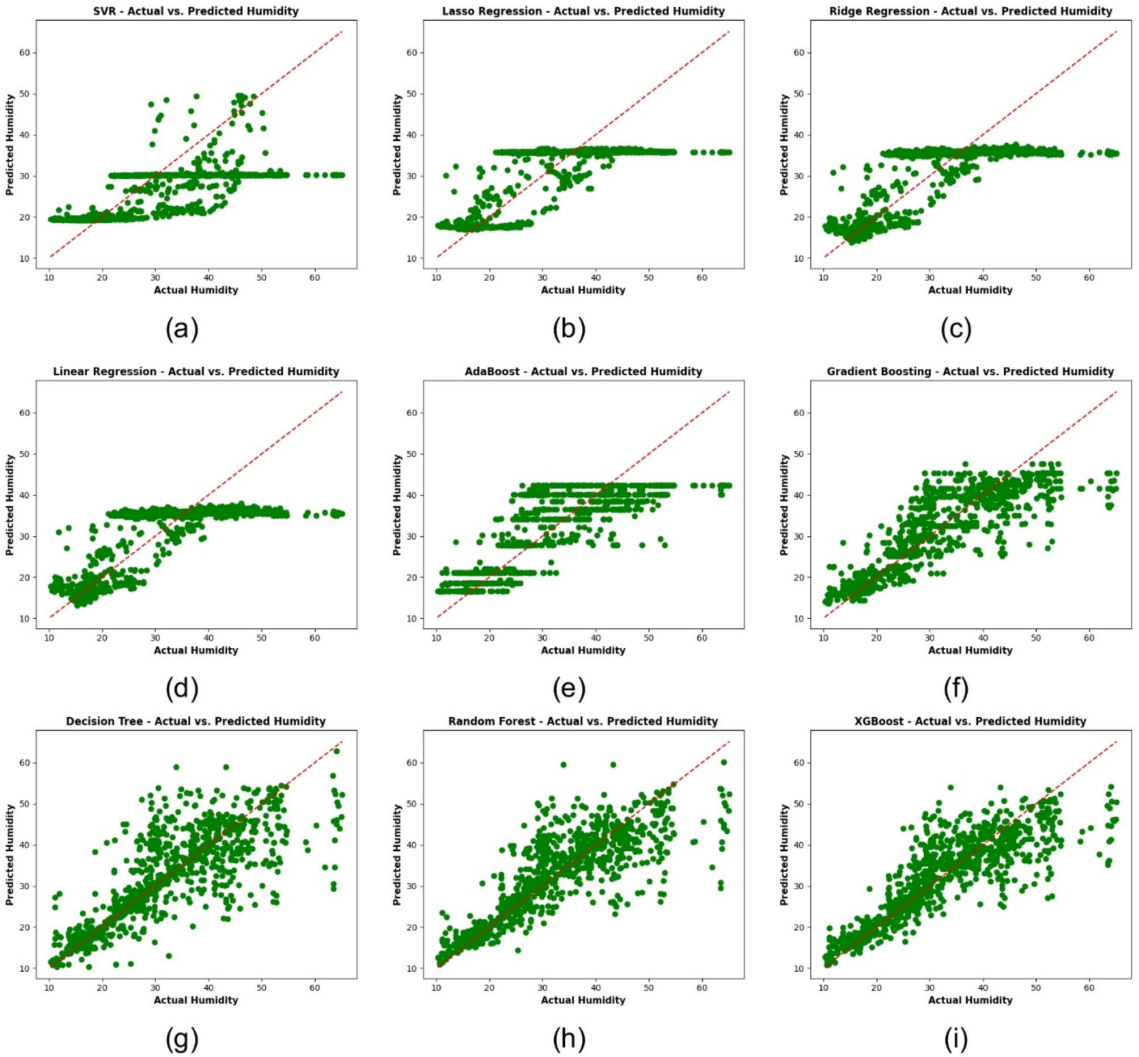
Scatter plots of predicted vs. actual humidity across various regression models: (**a**) SVR, (**b**) Lasso regression, (**c**) RR, (**d**) LR, (**e**) AdaBoost, (**f**) GB, (**g**) Decision tree, (**h**) RF, (**i**) XGBoost.

In Fig. 17, this study presents scatter plots of predicted versus actual humidity values for nine different regression models: Fig. 17a SVR, Fig. 17b Lasso Regression, Fig. 17c RR, Fig. 17d LR, Fig. 17e AdaBoost, Fig. 17f GB, Fig. 17g Decision Tree, Fig. 17h RF, and Fig. 17i XGBoost. The plots show how well each model reproduces the observed humidity, with lower deviations represented by closer points on the diagonal line. Two subplots create a visual representation and comparison to see how consistently each model is able to obtain the relationship between measured humidity and input features measured.

**Fig. 18:**
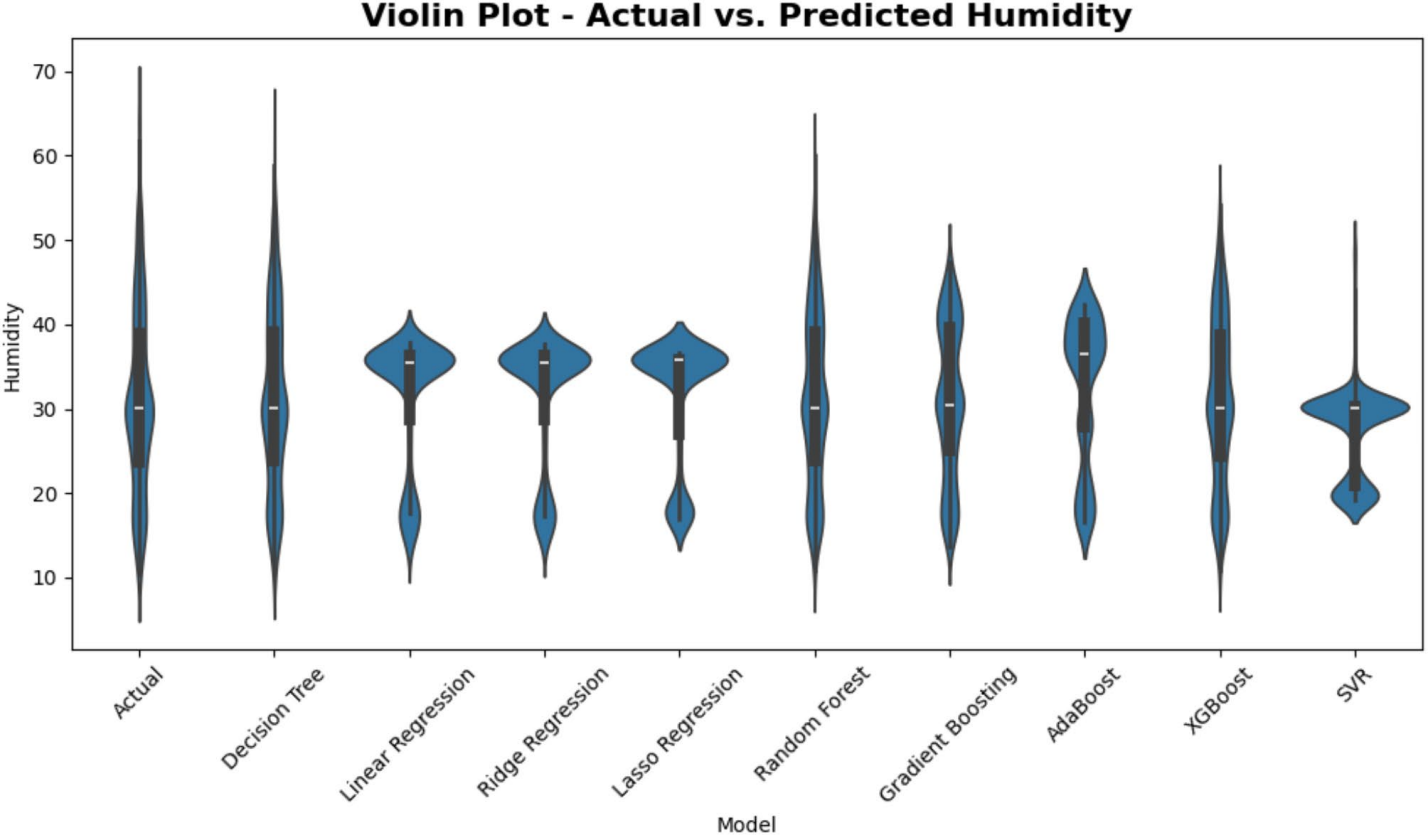
Violin plot of actual vs. predicted humidity across various regression models.

It also plots violins in Figure 18 showing the distribution of the actual humidity values against the predicted ones for different regression models. And each ‘violin’ holds a kernel density estimate from the data, showing where the values are more densely packed up — and how the values spread over the observed range. The final outputs of each model can give some comparison of the models about how accurately they replicate humidity conditions in reality, by comparing the shapes along with average lines of each model’s predicted distribution against the real humidity distribution.

### SHAP analysis for feature importance in XGBoost

This study used SHAP analysis based on feature importance to help interpret predictions generated by the XGBoost model. The SHAP values show how much each feature contributed to the predicted temperature and humidity for each input, providing insight into how the model arrived at its predictions.

**Fig. 19:**
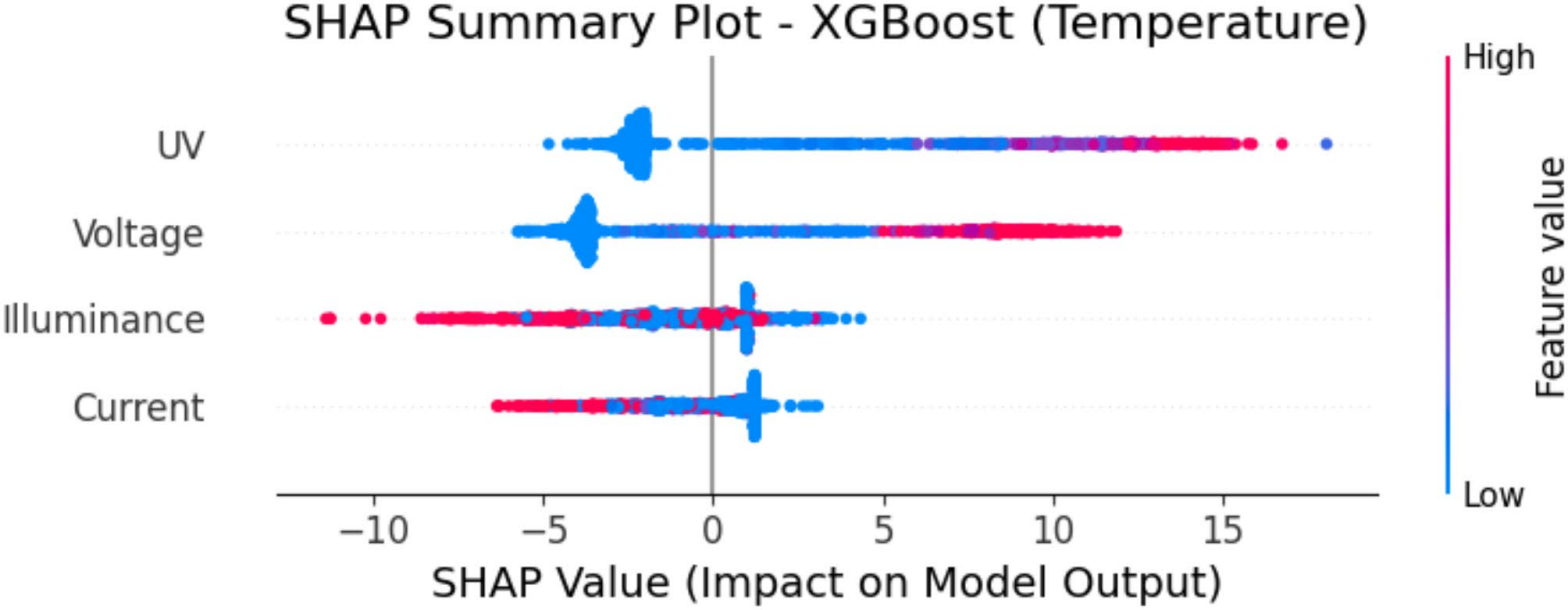
SHAP summary plot – XGBoost (temperature prediction).

Figure 19 presents the SHAP summary plot for temperature prediction using XGBoost. The plot illustrates the effect of each input feature on the model’s output. Features with a higher SHAP value have a more significant impact on the prediction. In this case, UV index and voltage appear to be the most influential variables, as indicated by their wider distribution and strong effect on the temperature forecast. The color gradient represents the feature value, where red indicates higher values and blue represents lower values.

**Fig. 20:**
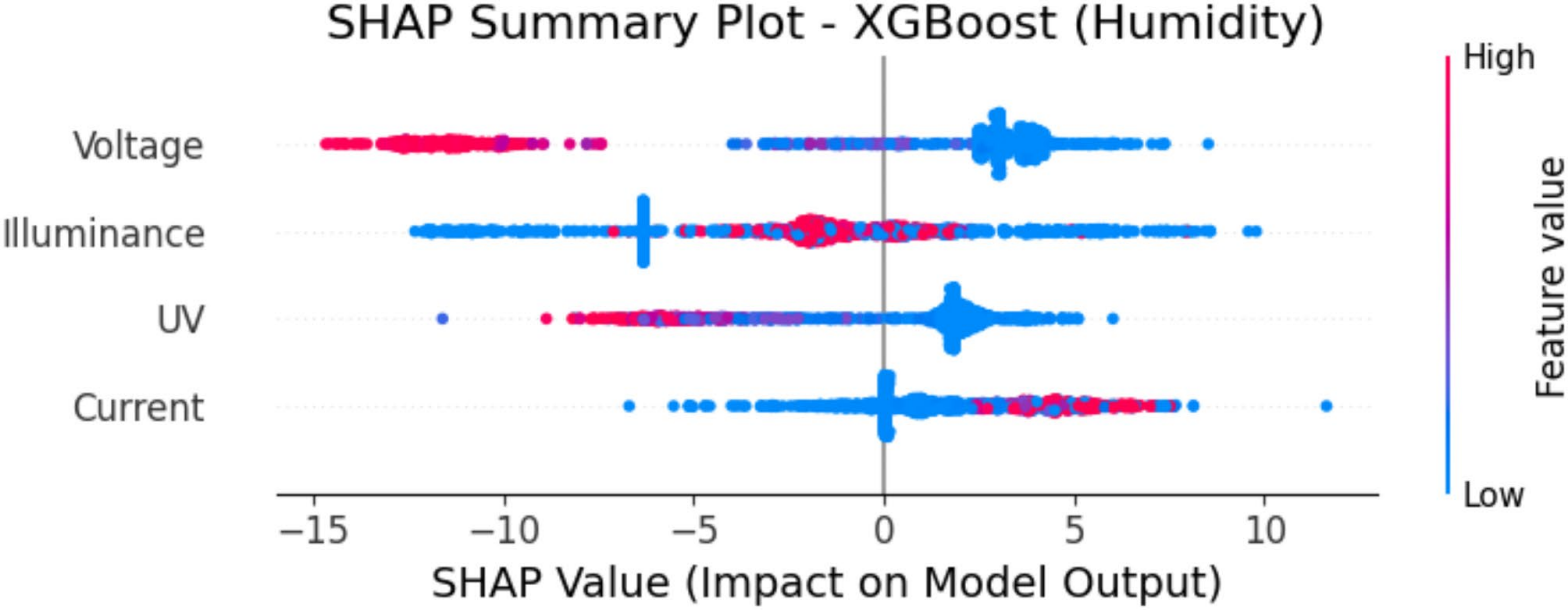
SHAP summary plot – XGBoost (humidity prediction).

Figure 20 illustrates the SHAP summary graphic for humidity prediction with XGBoost. The narrative emphasizes the comparative significance of many characteristics in forecasting humidity. Voltage and illuminance are the primary determinants, considerably influencing the model’s predictions. The distribution of SHAP values indicates that elevated voltage levels (red) significantly influence humidity estimates, whilst diminished illuminance values (blue) adversely affect the projected humidity levels.

## Future work

While this study offers a comparative review of several machine-learning models in the context of temperature and humidity prediction for PV locations, many future avenues can be addressed. Future work should explore various novel hybrid and deep learning methods such as LSTM-ALO, LSTM-INFO, RVFL-EROA, ANN-ERUN and ANN-RUNAO shown promise in time series forecasting. Moreover, including more environment factors could generate better robustness of the models and improvement in the prediction accuracy. The incorporation of metaheuristic optimization techniques (for example, Genetic Algorithms and Particle Swarm Optimization) could improve hyperparameter tuning, and signal decomposition methods (for example, Wavelet Transform and Empirical Mode Decomposition) could reduce data noise. Moreover, real-time ML models may be used for edge computing or cloud-based applications to provide continuous environmental monitoring and automated decision-making for PV systems. Future research should investigate transfer learning and domain adaptability across geographic regions, enabling models from one place to be applied in another with little retraining. Such comparison between data-driven ML models and physics-based one may further help understand model reliability and interpretability. Moreover, improving explainability via SHAP and Local Interpretable Model-Agnostic Explanations (LIME) will improve trust and transparency in ML-based forecast systems. Further work should also investigate multi-objective optimization techniques to maximize PV efficiency and minimize the cost of operations simultaneously. Large-scale validation on heterogeneous data from different PV farms is required to ensure model generalizability and real-world applicability. These perspectives will assist in the development of intelligent, data-based, and sustainable PV forecasting systems, contributing to reliable and efficient solar energy technologies.

## Conclusion

The paper presents a comprehensive analysis of several ML models used to forecast temperature and humidity in PV settings. Based on this investigation, it is clear that ensemble approaches, namely XGBoost and RF, have higher prediction ability when it comes to handling intricate environmental data. The models successfully captured complex patterns and connections in the information, resulting in improved accuracy and generalization for predicting both temperature and humidity. The findings indicate that sophisticated ensemble approaches are superior in addressing non-linearity and interactions in the data when compared to classic linear models and SVR. Moreover, the significant difference in performance between different models highlights the crucial role of selecting the appropriate model when implementing predictive systems in PV environments. By using powerful algorithms such as XGBoost, those involved may enhance the accuracy of environmental monitoring, eventually improving the effectiveness and dependability of solar systems. Given these discoveries, next investigations should examine the incorporation of other data sources, such as wind speed or solar radiation, in order to enhance the accuracy of forecast models. Furthermore, it is possible to explore sophisticated methods for optimizing hyperparameters and deploying ML models in real-time in operational PV systems. This research aims to improve the practical usability of these ML approaches. The knowledge acquired from this study establishes a basis for enhancing the performance of ML applications in renewable energy systems, hence promoting the development of more environmentally friendly and data-oriented energy solutions.

## Data Availability

The datasets used and/or analysed during the current study available from the corresponding author on reasonable request.

## Abbreviations

ANN: Artificial neural network
ANFIS: Adaptive neuro-fuzzy inference system
CA: Combined accuracy
DT: Decision tree
EMD: Empirical mode decomposition
GB: Gradient boosting
L1: Lasso regularization
L2: Ridge regularization
LIME: Local interpretable model-agnostic explanations
LR: Linear regression
MAE: Mean absolute error
ML: Machine learning
MLR: Multiple linear regression
PV: Photovoltaic
R^2^: Coefficient of determination
RF: Random forest
RMSE: Root mean squared error
RR: Ridge regression
SHAP: Shapley additive explanations
STL: Seasonal-trend decomposition
SVR: Support vector regression
SVM: Support vector machine
UV: Ultraviolet
XGBoost: EXtreme gradient boosting
